# Transformation of coffee-growing landscapes across Latin America. A review

**DOI:** 10.1007/s13593-021-00712-0

**Published:** 2021-08-31

**Authors:** Celia A. Harvey, Alyssa A. Pritts, Marie J. Zwetsloot, Kees Jansen, Mirjam M. Pulleman, Inge Armbrecht, Jacques Avelino, Juan F. Barrera, Christian Bunn, Javier Hoyos García, Carlos Isaza, Juana Munoz-Ucros, Carlos J. Pérez-Alemán, Eric Rahn, Valentina Robiglio, Eduardo Somarriba, Vivian Valencia

**Affiliations:** 1 Monteverde Institute, Apdo.69-5655, Monteverde, Puntarenas, Costa Rica; 2 Farming Systems Ecology Group, Wageningen University & Research, P.O. Box 430, 6700 AK Wageningen, The Netherlands; 3 Soil Biology Group, Wageningen University & Research, P.O. Box 47, 6700 AA Wageningen, The Netherlands; 4 Rural Sociology Group, Wageningen University & Research, Hollandseweg 1, 6706 KN Wageningen, The Netherlands; 5 The International Center for Tropical Agriculture (CIAT), Km 17 Recta Cali-Palmira, AA 6713, 763537 Cali, Colombia; 6 Departamento de Biología, Universidad del Valle, Calle 13 # 100-00 ed, 320 Cali, Colombia; 7 CIRAD, UMR PHIM, San José, Costa Rica; 8 PHIM, Univ Montpellier, CIRAD, INRAE, Institut Agro, IRD, Montpellier, France; 9 Program of Agriculture, Livestock and Agroforestry, CATIE, Turrialba, 7170 Costa Rica; 10 IICA, 2200 Coronado, San José, AP 55 Costa Rica; 11 Arthropod Ecology and Pest Management Group, Department of Agriculture, Society and Environment, El Colegio de la Frontera Sur, Carretera Antiguo Aeropuerto km 2.5, 30700 Tapachula, Chiapas Mexico; 12 University of Göttingen, Platz der Göttinger Sieben 5, 37073 Göttingen, Germany; 13 Parque Tecnológico de Innovación TECNiCAFÉ, Cra 17 # 48 N 18 Casa 53 Conjunto Cerrado Entrepinos, Popayán, Cauca Colombia; 14 Programa de Café para Solidaridad en Colombia, Solidaridad, Calle 43 N, °23-78 Manizales, Colombia; 15 School of Integrative Plant Science, Cornell University, 236 Tower Rd, Ithaca, NY USA; 16 Fundación Solidaridad Latinoamericana, Calle Evelio Lara No. 131-B, Ciudad del Saber, Ciudad de Panamá, Panamá; 17 World Agroforestry Centre (ICRAF), c/o CIP, Av. La Molina 1895, P.O Box 1558, 12 Lima, Peru

**Keywords:** Agroforestry systems, Certification, *Coffea arabica*, *Coffea canephora*, Coffee leaf rust, Deforestation, Intensification, Land-use change

## Abstract

In Latin America, the cultivation of Arabica coffee (*Coffea arabica*) plays a critical role in rural livelihoods, biodiversity conservation, and sustainable development. Over the last 20 years, coffee farms and landscapes across the region have undergone rapid and profound biophysical changes in response to low coffee prices, changing climatic conditions, severe plant pathogen outbreaks, and other drivers. Although these biophysical transformations are pervasive and affect millions of rural livelihoods, there is limited information on the types, location, and extent of landscape changes and their socioeconomic and ecological consequences. Here we review the state of knowledge on the ongoing biophysical changes in coffee-growing regions, explore the potential socioeconomic and ecological impacts of these changes, and highlight key research gaps. We identify seven major land-use trends which are affecting the sustainability of coffee-growing regions across Latin America in different ways. These trends include (1) the widespread shift to disease-resistant cultivars, (2) the conventional intensification of coffee management with greater planting densities, greater use of agrochemicals and less shade, (3) the conversion of coffee to other agricultural land uses, (4) the introduction of Robusta coffee (*Coffea canephora*) into areas not previously cultivated with coffee, (5) the expansion of coffee into forested areas, (6) the urbanization of coffee landscapes, and (7) the increase in the area of coffee produced under voluntary sustainability standards. Our review highlights the incomplete and scattered information on the drivers, patterns, and outcomes of biophysical changes in coffee landscapes, and lays out a detailed research agenda to address these research gaps and elucidate the effects of different landscape trajectories on rural livelihoods, biodiversity conservation, and other aspects of sustainable development. A better understanding of the drivers, patterns, and consequences of changes in coffee landscapes is vital for informing the design of policies, programs, and incentives for sustainable coffee production.

**Supplementary Information:**

The online version contains supplementary material available at 10.1007/s13593-021-00712-0.

**Contents**

- 1. Introduction
- 2. Methods
- 2.1 Land-use dynamics in coffee-growing regions
- 2.2. Potential ecological, social and economic consequences of ongoing landscape changes
- 2.3. A research agenda for understanding the drivers, patterns, and potential outcomes of land-use change and informing coffee sustainability policies and practice
- 3. Conclusions
- 4. Declarations
- 5. Literature Cited
- Acknowledgements

## Introduction

Coffee cultivation plays a vital economic, social, cultural, and environmental role in Latin America and shapes rural landscapes and livelihoods across the region. Latin America is a key region for coffee production, accounting for roughly 60% of the world’s coffee supply and 80% of the world’s Arabica coffee (*Coffea arabica*; ITC 2011). Millions of farmers, agricultural laborers, and other workers across the region depend on coffee production, purchasing, and processing for their livelihoods (Canet Brenes et al. 2016; CABI 2018). Coffee has consistently played an integral role in the region’s economy, and coffee exports continue to be an important source of income generation, though the overall contribution of coffee to national GDPs has declined in recent decades (CABI 2018). Since many of Latin America’s coffee-growing areas overlap with biodiversity hotspots (Jha et al. 2014), coffee cultivation practices (e.g., the type and use of shade trees, agrochemical use, soil conservation practices) can have variable and significant impacts on biodiversity conservation and the supply of ecosystem services, including carbon sequestration and water provision (Perfecto et al. 1996; Somarriba et al. 2004). The coffee sector therefore plays a critical role in efforts to improve farmer livelihoods, enhance biodiversity conservation, tackle climate change, and achieve sustainable development across Latin America.

In the coffee-growing regions of Mexico, Central America, and the Andean countries (referred to hereafter as “northern and Andean Latin America”), coffee production has historically involved the planting of Arabica coffee, a high-quality coffee that is grown in higher, cooler elevations. The majority of coffee farmers across this region are smallholders who cultivate small coffee plots (typically < 5 ha and often <2 ha), often in combination with annual crops (e.g., maize, beans), fruit and timber trees, small-scale livestock production, or small areas of forest (Méndez et al. 2010; Bacon et al. 2017; Harvey et al. 2017; Panhuysen and Pierrot 2020). Consequently, coffee landscapes are typically diverse landscape mosaics composed of coffee fields interspersed with other land uses. Arabica coffee is grown under a wide range of management types from traditional rustic systems where coffee bushes are planted under heavily thinned natural forest and few inputs are used, to specialized shade systems where shade trees and inputs are carefully managed, to intensive systems with high densities of coffee bushes, little or no shade, and heavy use of agrochemical inputs (Moguel and Toledo 1999; Perfecto et al. 2019). Depending on the type, diversity, and density of their shade canopy, coffee agroforestry systems can provide fruit, firewood, timber, and other goods to farmers (Rice 2008); serve as critical habitat, resources, and landscape connectivity for biodiversity conservation (Perfecto et al. 1996; Moguel and Toledo 1999; Somarriba et al. 2004; Valencia et al. 2016); store significant carbon stocks and thereby contribute to climate mitigation (e.g., Haggar et al. 2013; Vaast et al. 2016); enhance the resiliency of agricultural landscapes and help farmers adapt to climate change (Eakin et al. 2014; Harvey et al. 2017) and provide valuable ecosystem services (such as water provision, soil conservation, pollination, habitat for biodiversity, fruit and firewood provision) that underpin rural livelihoods (Jha et al. 2011; Cerda et al. 2017).

Coffee production across northern and Andean Latin America has historically gone through periods of expansion and contraction in response to market supply and demand, climatic events, pest and disease outbreaks, and volatile coffee prices (Flores et al. 2002; Blackman et al. 2007). For example, in the 1970s and 1980s, the combination of neoliberal policies, growing global demand for coffee, and the need to prevent the spread of coffee leaf rust (*Hemileia vastatrix)* led to the rapid intensification of coffee landscapes, with many shaded coffee farms being converted to low-shade or open-sun systems with high agrochemical inputs and densely planted coffee bushes (Perfecto et al. 1996, 2019; Rice 1999). However, for the last two decades, the biophysical changes in coffee-growing landscapes have been particularly pronounced and rapid. The widespread changes in coffee-growing regions reflect a combination of interacting stressors and shocks. Global coffee prices are volatile but have followed a continued downward trend since 2016 (ICO 2019). In September 2018, coffee prices were the lowest in 12 years (Amico et al. 2020). The prolonged period of low prices, coupled with rising labor and input costs, threatens the viability of coffee farming in the region (CABI 2018; ICO 2019; Panhuysen and Pierrot 2020). The COVID-19 pandemic is placing additional stress on coffee production, as sanitary measures implemented in response to the pandemic are affecting the costs of production, reducing the availability and cross-border movement of workers who harvest coffee, and disrupting field visits by extension services (Aquino 2020; Guido et al. 2020; Panhuysen and Pierrot 2020). At the same time, the coffee sector is facing significant challenges from climate change, as rising temperatures, changing precipitation patterns, and more frequent and intense extreme weather events (e.g., severe droughts, hurricanes, and flooding) reduce yields and quality, increase pest and disease outbreaks, and change the suitability of areas for coffee growing (Bunn et al. 2015; Läderach et al. 2017; Harvey et al. 2018). Climate change is expected to significantly reduce the area available for coffee production in Latin America in the future unless adaptation measures are put in place (Bunn et al. 2015; Läderach et al. 2017).

The region’s coffee production has also been profoundly affected by severe outbreaks of coffee leaf rust (a disease caused by the *Hemileia vastatrix* fungus) which results in heavy yield losses (Avelino et al. 2015; Avelino and Anzueto 2020). Leaf rust outbreaks have swept across northern and Andean Latin America, moving from Colombia (2008 to 2011), to Central America and Mexico (2012 onwards) to Peru and Ecuador in 2013. In Central America alone, coffee leaf rust reduced yields by 10–55% with regard to pre-rust levels (Amico et al. 2020) corresponding to an estimated 515 million USD in losses (ICO 2014), led to widespread food insecurity and malnutrition of smallholder coffee farmers and laborers (Avelino and Anzueto 2020), caused massive unemployment, and significantly increased migration to North America (Dupre 2018). Although coffee production started to recover from 2014 onwards due to the implementation of expensive management measures (such as coffee plant renovation and increased use of fungicides), the disease continues to hamper production in the region (Avelino and Anzueto 2020).

**Fig. 1:**
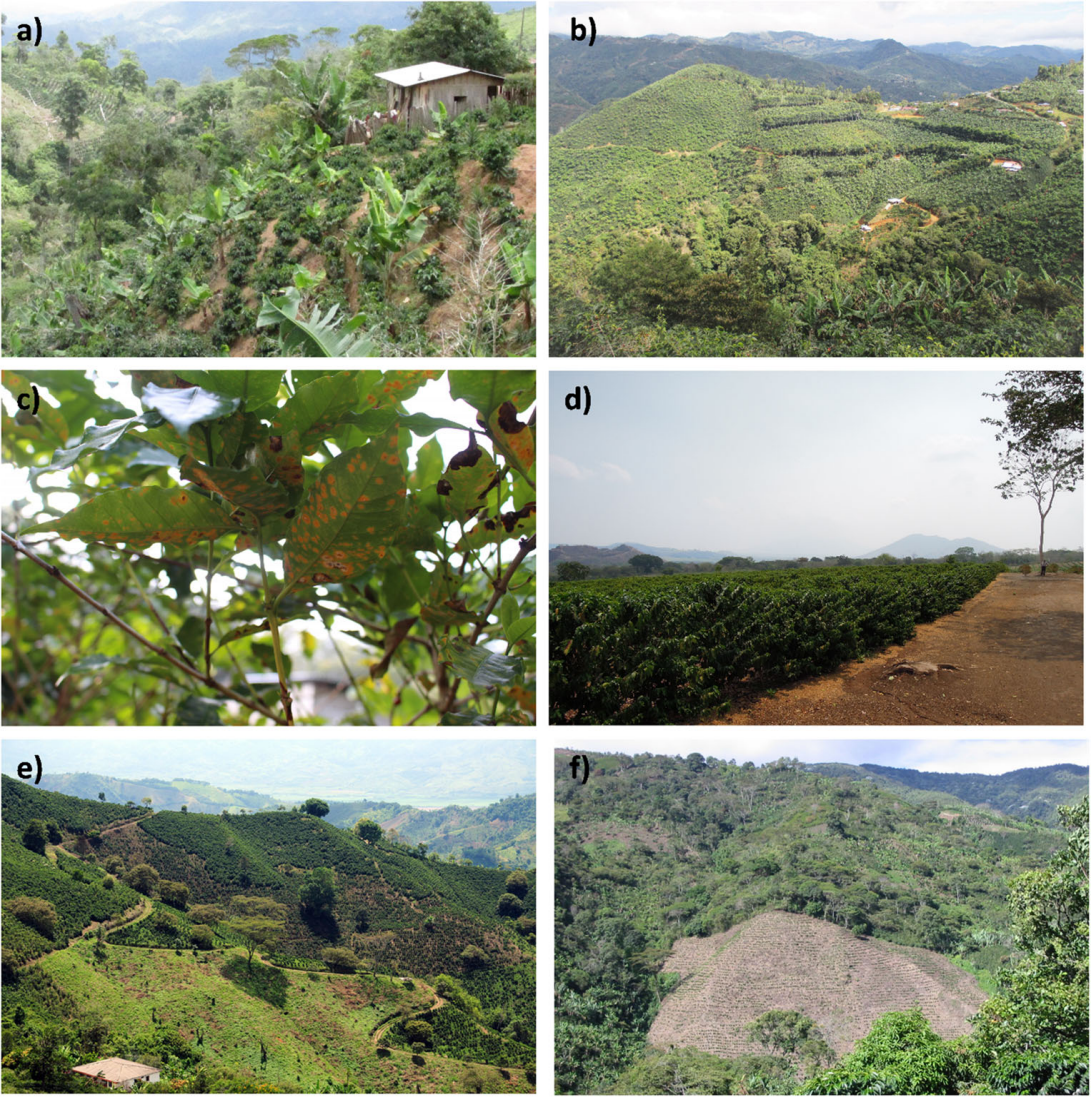
Photographs illustrating the current land use and ongoing transformation of coffee farms and landscapes across Latin America: **a**) a typical smallholder coffee farm in Yoro, Honduras, where coffee is grown in small plots under a sparse canopy of bananas and shade trees; **b**) a landscape dominated by intensive coffee production with highly managed shade in Los Santos, Costa Rica; **c**) coffee leaf rust, a disease which has caused widespread damage to coffee plantations and led farmers to replace traditional coffee varieties with disease-resistant varieties; **d**) an example of an intensified coffee farm in the lowlands of El Salvador, with coffee grown in open sun and with irrigation; **e**) a landscape in Colombia’s coffee zone where some coffee fields have been converted to pasture and other agricultural land uses; and **f**) a landscape in Risaralda, Colombia, where coffee agroforests and forests have been cleared to establish new coffee. Photo credits: Milagro Sandoval (**a**), Jose Mario Cardenas (**b**), Alyssa Pritts (**c**), Jacques Avelino (**d**), ©2009CIAT/Neil Palmer (**e**), Inge Armbrecht (**f**).

The confluence of low prices, unfavorable climatic condition, coffee leaf rust, increasing production costs, and other stresses has led to profound and unprecedented biophysical changes to coffee farms and landscapes across northern and Andean Latin America, transforming how and where coffee is grown (Figure 1). In some coffee-growing areas, coffee fields have been abandoned and replaced by pastures, other agricultural crops, or other land uses (e.g., Haggar et al. 2013; Babin 2020), changing the composition and spatial configuration of coffee landscapes. In other regions, coffee is expanding into new areas, sometimes leading to deforestation (e.g., Blackman et al. 2005). Hundreds of thousands of hectares of coffee are also being renovated with high-yielding coffee varieties that are resistant to coffee leaf rust, and/or being put under intensified conventional management practices (including a reduction in shade tree cover and greater use of agrochemicals), leading to changes in both coffee productivity and the structure and composition of coffee landscapes. In other landscapes, there is an expansion in the area of coffee produced following voluntary sustainability standards that reward, among other measures, the maintenance or increase of shade density and diversity, forest conservation, soil conservation, and responsible use of pesticides and other agrochemicals (Milder et al. 2014; Lernoud et al. 2018). While some of these changes in coffee farms and landscapes have been documented previously in specific regions (e.g., Guhl 2008; Jha et al. 2014; Hite et al. 2017), there is very little comprehensive information on the extent, magnitude, dynamics, and consequences of landscape change across the Latin American region. The limited information on the ongoing transformation of coffee landscapes is concerning given the pervasiveness of biophysical changes and their potential to significantly affect the socioeconomic and ecological sustainability of coffee landscapes and livelihoods.

The objective of this paper is to synthesize biophysical changes in coffee farms and landscapes across northern and Andean Latin America, explore potential consequences of these changes, and identify key research needs for better understanding of the dynamics and consequences of landscape transformations. We focus our review on coffee-growing regions of Mexico, Central America, and the Andean countries of Colombia, Peru, and Ecuador, as these coffee-growing regions share similar coffee production systems. Using expert knowledge and available literature, we highlight the major land-use trends occurring in coffee landscapes in response to coffee leaf rust, low prices, climatic change, increasing production costs, and other drivers, and explore some of the potential consequences of these changes on the social, economic, and environmental sustainability of coffee systems. We also identify key elements of a research agenda that could enable a more comprehensive understanding of the drivers, dynamics, and impacts of different landscape trajectories and how these trajectories could either contribute to — or detract from — current efforts to promote more sustainable coffee landscapes. This information is critical for informing the development of public and private sector strategies, programs, and incentives to promote a more sustainable coffee sector (e.g., Millard 2017; ICO 2019; Bager and Lambin 2020; Panhuysen and Pierrot 2020).

## Methods

We used a two-pronged approach to assess the current state of knowledge on ongoing landscape transformations in coffee-growing landscapes and identify key research gaps. First, we organized an expert workshop at the Alliance of Bioversity International and the International Center for Tropical Agriculture (CIAT) in Cali, Colombia (February 17–20, 2020), with representatives from coffee institutions, development NGOs, agricultural extension agencies, farmer organizations, agricultural research centers, and academia. The experts possessed long-term experience and deep knowledge of one or more coffee regions in Latin America. Participants included experts from Mexico, Costa Rica, Nicaragua, Peru, and Colombia, but many experts also had experience in other countries across the region. The participants represented a wide range of expertise, including phytopathology, agroecology, entomology, plant physiology, soil science, conservation biology, climate change, market value chains, and political ecology. During the workshop, the participants discussed changes in the coffee sector and drivers of coffee landscape change, identified biophysical changes that had occurred (or were ongoing) within different coffee regions during the last two decades, and explored the known socioeconomic and ecological impacts of these landscape-level changes. They also identified knowledge gaps and research needs.

Second, we conducted a detailed literature review (primarily of scientific literature, but also of grey literature from key national-level coffee organizations, national research centers, and the International Coffee Organization) to find examples of the key landscape trajectories identified in the workshop and to identify the main knowledge gaps. Our literature review centered on publications on landscape change in coffee-growing regions during the last 20 years (2000–2020); however, because there is often a delay between research and publication, some of the publications included also provide information on landscape and management changes observed in the late 1990s.

In our focus region (northern and Andean Latin America), coffee is a major land use, covering more than 2.8 million ha of land (Table 1). Most of the farmers cultivate Arabica coffee, which represents ~94% of the coffee area grown, in mountainous areas, often on steep slopes. Coffee production is the livelihood of an estimated 1.68 million farmers in the region, the majority of whom are smallholders (Table 1). Coffee has consistently played an integral role in the region’s economy, serving as a critical source of income generation and employment for millions of people (in coffee production, harvesting, processing, and retail) and contributing between 0.3 and 3.7% of countries’ gross domestic product (Table 1).

**Table 1:** An overview of the importance of coffee in different countries within northern and Andean Latin America, including area planted with coffee, the number of coffee farmers, the contribution of coffee to national exports or national gross domestic product, and the number of people dependent on coffee for their livelihoods (including farmers, coffee pickers and laborers, and jobs related to the coffee sector), with “NA” indicating data were not available. Years in parentheses indicate the years to which the data refer. ^1^(USDA FAS 2019); ^2^(USDA FAS 2020b); ^3^(ICAFE 2020); ^4^(Federación Nacional de Cafeteros de Colombia 2019); ^5^(USDA FAS 2020a); ^6^(IDH 2019); ^7^(Garcia et al. 2014); ^8^(USDA FAS 2020a); ^9^(USDA FAS 2020a); ^10^(Wiegel et al. 2020); ^11^(Consejo Salvadoreño del Café 2020); ^12^(Fernandez-Kolb et al. 2019); ^13^(USAID 2017); ^14^(USDA FAS 2020a); ^15^(Bunn et al. 2019); ^16^(Anacafé 2019); ^17^(USDA FAS 2020a); ^18^(Bunn et al. 2018); ^19^(USDA FAS 2020a); ^20^(Secretaría de Agricultura y Desarrollo Rural 2018); ^21^(Ellis et al. 2010); ^22^(USDA FAS 2020a); ^23^(Quiroga et al. 2020); ^24^(Escobedo Aguilar et al. 2017); ^25^(Ministerio de Desarrollo Agropecuario (MIDA), 2018); ^26^(Favovich 2020); ^27^(USDA FAS 2020a); ^28^(Banco Central de Reserva del Peru 2020); ^29^(León-Carrasco 2020)

Country	Area under coffee (ha)			Coffee production in 2019 (in metric tons)^1^		Coffee livelihoods		Economic importance		
	Area planted with Arabica	Area planted with Robusta	Total area planted with coffee	Total Arabica production	Total Robusta production	# of coffee farmers	% of coffee farmers who are smallholders	% of gross domestic product	% of export revenues	# of people dependent on coffee for their livelihoods
Costa Rica	93,697 (2018)^2^	0^1^	93,697 (2018)^2^	76.6	0	38,804 (2018/19)^2^	91.4% (2018/19)^3^	0.27% (2018/19)^2^	2.5% (2018/19)^3^	NA
Colombia	853,700 (2019)^4^	0^5^	853,700 (2019)	832.2	0	540,000 (2019)^5^	95% (2019)^6^	3.4% (2019)^6^	7% (2019)^6^	2,000,000 (2014)^7^
Ecuador	NA	NA	48,097 (2019)^8^	8.4	6.9	60,000 (2019)^8^	>95% (2019)^8^	NA	NA	NA
El Salvador	137,000 (2019)^9^	0 (2019)	137,000 (2019)^9^	39.2	0	23,751 (2020)^10^	~85% (2020)^11^	0.56% (2016)^12^	2.6% (2015)^13^	45,000 (2019)^12^
Guatemala	274,500 (2019)^14^	30,500 (2019)^14^	305,000 (2019)^14^	211.2	15	>122,000 (2019)^15^	98% (2019)^15^	1.04% (2016)^15^	24% (2019)^16^	~500,000 (2019)^15^
Honduras	312,000 (2019)^17^	0	312,000 (2019)^17^	450.9	0	~110,000 (2019)^18^	87% are small and medium holders (2020)^17^	3.7% (2015)^18^	NA	~1,000,000 (2019)^18^
Mexico	500,000 (2019)^19^	80,000 (2019)	580,000 (2019)	186	27	481,000 (2010)^21^	~48% (2017)^13^	0.66% (2018)^20^	1.34% (2018)^20^	Up to 1 million jobs (2010)^21^
Nicaragua	137,900 (2019)^22^	2100 (2019)^22^	140,000 (2019)^22^	156	3	45,000 (2012)^10^	97% (2017)^23^	2% (2017)^24^	8.3% (2015)^13^	332,000 (2017)^22^
Panama	NA	NA	9634 (2018)^25^	75	0	3166 (2018)^23^	NA	0.4% (2020)^26^	2%^23^	NA
Peru	375,000 (2019)^27^	0	375,000 (2019)^27^	4480	0	223,482 (2019)^10^	59% (2017)^13^	0.64% (2019)^28^	1.46% (2019)^28^	2,000,000 (2020)^29^

We excluded Brazil from our analysis because its coffee production is distinct from that of the target region (Canet Brenes et al. 2016; Volsi et al. 2019). Contrary to its production in northern and Andean Latin America where coffee is grown at high elevations and shade trees are common, Brazilian coffee is primarily grown under full sun, on less steep slopes, and is intensively produced, often with mechanization and irrigation (Jha et al. 2014; Volsi et al. 2019). We also excluded Venezuela, Bolivia, and Panama from the analysis, due to the limited coffee production occurring in these countries and the relatively limited information on their coffee sectors.

## Land-use dynamics in coffee-growing regions

Our analysis suggests that there are at least seven major types of farm and landscape-level changes across northern and Andean Latin America that are reshaping the spatial configuration and makeup of coffee-growing landscapes in divergent ways (Table 2). These trends in land-use change are not necessarily mutually exclusive and may occur simultaneously in the same landscape or region. In addition, two of the trends (conventional intensification and increased in the area of coffee produced under voluntary sustainability standards) affect shade levels and agrochemical use in opposite ways.

**Table 2:** A summary of biophysical trends in coffee farms and landscapes across northern and Andean Latin America, with examples of identified trends at the national and local levels. (Additional details on the papers highlighted here can be found in Supplementary Table 1).

Trend	Papers highlighting these trends
1. Replacement of traditional Arabica varieties by introgressed varieties that are resistant to coffee leaf rust	*National-level examples*: Colombia (van der Vossen, 2005); Ecuador (INEC 2019); Guatemala (Bunn et al. 2019); Honduras (Avelino and Anzueto 2020; Wiegel et al. 2020); Mexico (Amico et al. 2020; USAD FAS 2020h); Peru (Romero 2020) *Case studies:* Copán, Honduras (Ward et al. 2017); La Sepultura, Mexico (Valencia et al. 2018)
2. Conventional intensification of coffee production, involving a reduction in shade levels, increased use of agrochemicals and greater density of coffee bushes	*National-level examples:* Costa Rica (Jha et al. 2014); Colombia (Jha et al. 2014); El Salvador (Blackman et al. 2012; Jha et al. 2014); Guatemala (Jha et al. 2014, Bielecki and Wingenbach 2019); Nicaragua (Jha et al. 2014); Mexico (Amico and Paz-Pellat 2018) *Case studies*: La Sepultura, Chiapas, Mexico (Valencia et al. 2018); San Martin, Peru (Jezeer et al. 2019)
3. Abandonment of coffee fields and conversion of coffee plots to other land uses	*National- level examples:* Colombia (Rueda and Lambin 2013a, 2013b; Portafolio 2018); Guatemala (Schmitt-Harsh 2013; Baumeister 2017); Peru (USDA FAS 2020a) *Case studies*: Municipality of Zozocolco in Veracruz, Mexico (Ellis et al. 2010); Emiliano Zapata in Central Veracruz, Mexico (Hausermann 2014); Central Veracruz, Mexico (Hausermann 2014); Southern Guatemala (Haggar et al. 2013); The Chinantla subregion of Sierra Norte de Oaxaca, Mexico (Hite et al. 2017); Veracruz, Mexico (Eakin and Webbe 2009); Agua Buena district, Costa Rica (Babin 2020); Turrialba, Costa Rica (Bosselmann 2012)
4. Expansion of coffee into forested areas, leading to deforestation	*National-level examples*: Honduras (Bunn et al. 2018); Amazonian regions of Ecuador and Peru (Somarriba and López Sampson 2018); Guatemala (Bunn et al. 2019) *Case studies*: The San Martin region of Peru (Marquardt et al. 2019); Amazonian departments in Peru (Ganzenmuller and Castro Nuñez 2019), The transboundary Trifinio region which spans Honduras, Guatemala and El Salvador (Schlesinger et al. 2017); Municipalities of El Provenir, Ángel Albino Corzo, Siltepec and the upper areas of La Concordia in Chiapas, Mexico (Covaleda et al. 2014), La Sepultura, Chiapas, Mexico (Valencia et al. 2018), Chiapas, Mexico (Cortina-Villar et al. 2012)
5. Introduction of Robusta into new areas where coffee was previously not grown	*National-level examples*: Guatemala: (Nicholson and Menchu 2018; VOA 2018), Nicaragua (VOA 2018; Pretel 2018), Mexico (Aceves Navarro et al. 2018), Colombia (Portafolio 2017) *Case studies*: The Autonomous Region of the South Atlantic of Nicaragua (Nicholson and Menchu 2018), Nueva Guinea, Nicaragua (Bjørge 2017), The Pacific slope of the Sierra Madre mountain range, Mexico (Amico et al. 2020), Southern Chiapas, Mexico (Barrera 2016)
6. Urbanization of coffee areas	*National-level examples*: Costa Rica (Filho et al. 2008, Jha et al. 2014), El Salvador (Blackman et al. 2012) *Case studies*: Veracruz, Mexico (Hausermann 2014); Pereira, Colombia (Portafolio 2018)
7. Increase in the coffee area grown under voluntary sustainability standards, with related changes in shade levels and/or on-farm forest cover	*Case studies*: Santander, Colombia (Rueda and Lambin 2013a, Rueda et al. 2015), Costa Rica, Guatemala and Nicaragua (Haggar et al. 2015)

The first landscape trend is that coffee farmers are actively changing the coffee varieties they grow, replacing traditional Arabica varieties (such as Bourbon or Typica) with high-yielding, introgressed coffee varieties (e.g., Catimores or Sachimors) that have been bred to be resistant to coffee leaf rust (Avelino and Anzueto 2020). Following the devastating coffee leaf rust outbreaks, national governments, coffee institutions, and the coffee industry have made concerted efforts to restore production by replanting affected plantations with resistant varieties, distributing resistant coffee plants, and providing technical support, agricultural inputs, and credit schemes to help cover farmers’ renovation costs (Valencia et al. 2018; Amico et al. 2020; Wiegel et al. 2020). For example, in response to the 2008 outbreak in Colombia, the National Federation of Coffee Growers began a national campaign, “Colombia sin roya” (or “Colombia without coffee leaf rust”), that replanted an estimated 45% of the country’s total coffee area with resistant Castillo cultivars (van der Vossen et al. 2015). In Honduras, renovation efforts increased the country’s area of coffee planted with resistant cultivars from 40 to 62%, though some of these so-called resistant cultivars (e.g., ‘Lempira’) have recently been shown to be losing resistance (Avelino and Anzueto 2020). In contrast, renovation in El Salvador has been much slower and more than 50% of the coffee area is still planted with susceptible varieties (Avelino and Anzueto 2020). Although there are no statistics on the full extent and distribution of resistant cultivars across the region (see Table 2 for available country-level information), many hundreds of thousands of hectares of affected coffee plantations have been replanted with resistant cultivars over the last decade. This large-scale replanting has transformed the structure and composition of many coffee landscapes and, in some cases, has reduced the extent and diversity of shade trees within coffee fields, simplifying landscape structure (Jha et al. 2014; Perfecto et al. 2019).

A second trend is that many coffee farmers are continuing to intensify the management of existing Arabica coffee fields in an effort to obtain higher yields, reduce labor costs, and control pest and disease outbreaks (Table 2). The “conventional intensification” of coffee production involves reducing, simplifying, or eliminating shade and increasing the planting density of coffee plants and the use of pesticides, fertilizers, and fungicides (Perfecto et al. 1996, 2019; Rice 1999; Guhl 2008). From the 1970s onwards, there has been a steady loss of diverse shade-grown coffee systems and their replacement by simplified shade systems or sun-grown coffee, with nearly 50% of shade coffee farms in Latin America converted to low-shade systems between 1970 and 1990 (Perfecto et al. 1996, 2019; Rice 1999; Jha et al. 2014). Shade-grown coffee systems are continuing to be lost in certain countries. For example, from 1996 to 2012, the percent of coffee area under traditional, diverse shade fell in El Salvador (from 92 to 24%), Nicaragua (from 55 to 25%), Guatemala (from 45 to 40%), and Costa Rica (from 10 to 0%; Jha et al. 2014 supplementary materials). However, during the same time period, shade levels in Colombia stayed more or less stable (~30%), and the percent of coffee under shade increased in Honduras (from 15 to 35%) and Mexico (from 10 to 30%; Jha et al. 2014, supplementary materials). In Costa Rica, almost all coffee is now grown either under simplified shade systems (in which trees are frequently pruned) or in open sun (Jha et al. 2014) with intensified management (Blackman and Naranjo 2012). Comprehensive data on the extent and distribution of intensified coffee production for the region, especially information on the density, composition and diversity of shade trees, and levels of agrochemical use, are either lacking or out of date (e.g., Rice 1999). In some landscapes, the rapid shift from high-yielding resistant varieties is leading to further intensification of coffee production, as farmers often plant the new varieties under little or no shade and apply heavy doses of agrochemicals (Perfecto et al. 2019; Amico et al. 2020). For example, smallholder farmers in both Guatemala (Bielecki and Wingenbach 2019) and in Chiapas, Mexico (Valencia et al. 2018) who previously produced organic coffee under diversified, dense shade, have recently switched to resistant varieties and started applying synthetic fertilizers and pesticides in an attempt to control pests and diseases and increase yields.

In regions where coffee production is increasingly difficult or unprofitable, a third trend is that coffee farmers are either abandoning their coffee plantations or converting some or all of their coffee fields to other land uses (Table 2). For many smallholder coffee farmers, coffee farming is no longer economically viable, as low coffee prices make it difficult to cover the high costs of labor, fertilizer, and other inputs needed to sustain coffee production (Blackman et al. 2005; van der Vossen 2005; Panhuysen and Pierrot 2020). Rising temperatures and coffee leaf rust outbreaks have also impacted coffee production, reducing yields and farmer incomes even further (ICO 2019; Panhuysen and Pierrot 2020). In these circumstances, many farmers are abandoning their coffee or converting them to other land uses in an attempt to restore farm productivity. For example, 42% of the coffee farmers in the Chinantla subregion of the Sierra Norte de Oaxaca, Mexico, abandoned their coffee agroforests or converted them to another land use from 1990 to 2010, due to low coffee prices, low yields, and other stressors (Hite et al. 2017). Similarly, the area under coffee in Turrialba, Costa Rica, decreased 7% annually from 2000 to 2009, as farmers replaced coffee with crops such as vegetables or sugarcane (Bosselmann 2012). In the community of Emiliano Zapata in Central Veracruz, Mexico, coffee farmers have converted a portion of their farm to sugarcane and also planted lime trees to diversify their income sources (Hausermann 2014). In some low-elevation regions of Peru and Colombia, the combination of declining coffee prices and disease outbreaks has led farmers to replace some or all of their coffee plantations with the cultivation of illegal crops such as coca leaves, which are more profitable (Rettberg 2010) or temporarily abandon coffee production and move to coca-producing areas (Grisaffi and Farthing 2021). Other examples include the conversion of coffee fields to sugarcane production (Tucker et al. 2010; Bosselmann 2012; Hausermann 2014), rubber (Haggar et al. 2013), cacao (Marquardt et al. 2019), fruit trees (Hausermann 2014), vegetable production (Bosselmann 2012), pastures (e.g., Ellis et al. 2010; Haggar et al. 2013; Babin 2015), and secondary forests (Eakin and Webbe 2009; Hite et al. 2017). While the abandonment and conversion of coffee to other land use are widespread and have gained significant media attention (e.g., Nicholson 2014; Semple 2019; Terazono et al. 2019), details on the extent and distribution of coffee abandonment and conversion, and how the overall structure and composition of coffee landscapes is changing, are generally lacking.

A fourth trend is that while the area under coffee is contracting in many regions, coffee production is also expanding into new areas previously under forest (Table 2), driven in part by the growing local and global demand for coffee (ICO 2019). Although there is surprisingly little information on the extent to which coffee is driving deforestation (Panhuysen and Pierrot 2018), there is evidence that coffee expansion is contributing to deforestation in certain regions, including the Chiapas and Oaxaca regions of Mexico (Blackman et al. 2005; Covaleda et al. 2014; Valencia et al. 2018), the Amazonian regions of Ecuador and Peru (Ganzenmuller and Castro Nuñez 2019; Marquardt et al. 2019), and the Trifinio transboundary area of El Salvador, Guatemala, and Honduras (Schlesinger et al. 2017). For example, in the Department of San Martin, Peru, the area under Arabica coffee production tripled to an estimated 102,101 ha between 1995 and 2010, largely at the expense of primary forest (Marquardt et al. 2019). The encroachment of coffee on forest areas is expected to intensify in the future, as optimal locations for coffee production will move up in elevation under climate change (Baca et al. 2014; Bunn et al. 2015). Since many Arabica coffee-growing regions are adjacent to forests or protected areas (e.g., in El Salvador, 72% of the protected areas are within a 10-km radius of coffee-growing areas; Jha et al. 2011), any upward shifts in coffee production could encroach on remaining forest areas and lead to biodiversity loss, if these areas are not adequately protected.

The fifth shift in coffee-landscapes is the recent introduction of Robusta coffee (*Coffea canephora*) in some countries including in areas that have no coffee-growing history. With the exception of Mexico and Guatemala, where Robusta coffee has been grown for decades on a limited scale, coffee production in northern and Andean Latin America has historically centered on the planting of Arabica coffee. To protect their reputations as providers of high-quality (Arabica) coffee, Costa Rica, Honduras, and Nicaragua have historically banned the production of Robusta coffee (Pretel 2018) and other countries (such as Colombia) have strongly discouraged Robusta production (Nicholson and Menchu 2018). Some of these Robusta bans have been recently lifted (in 2013 in Nicaragua, and 2018 in Costa Rica; Pretel 2018), opening up the door for Robusta production, though there is still resistance among some of the coffee-growing organizations. Robusta coffee is a high-yielding species that generally produces lower quality (and lower value) coffee, is less susceptible to coffee leaf rust, and able to tolerate hotter temperatures, a characteristic that is increasingly desirable given the rising temperatures across the region (Bunn et al. 2015). As a result, Robusta coffee can be grown in lower elevations that are marginal for Arabica production. While it is unclear to what extent Robusta will spread across the region, recent news reports indicate that farmers in Mexico, Nicaragua, and Guatemala are being encouraged to plant Robusta in new areas (Nicholson and Menchu 2018; VOA 2018). For example, in Mexico, the government is supporting the establishment of an additional 20,000 ha of Robusta coffee in the southern and southeastern states of Chiapas, Veracruz, and Tabasco (Aceves Navarro et al. 2018). This expansion is mainly into lowland areas where coffee has previously not been grown, leading to deforestation, but in some areas of Chiapas, Robusta coffee is being planted in areas that were previously under Arabica coffee (Barrera 2016; Amico et al. 2020). In Nicaragua, the coffee industry started testing the suitability of Robusta coffee in the Autonomous Region of the South Atlantic (Bjørge 2017; Nicholson and Menchu 2018) in 2005 and had planted Robusta on nearly 900 hectares by 2016 (Bjørge 2017). A recent Nicaraguan law allows the production of Robusta in all regions of Nicaragua lower than 400 masl and in those located more than 30 km from Arabica plantations (Gonzalez 2016), setting the stage for further Robusta expansion. In Colombia, Robusta coffee is being tested in different regions of the country where Arabica is not grown, but has not yet been planted at scale (Portafolio 2017).

A sixth trend is the increasing urbanization of coffee landscapes, especially near major cities. A key example is the Central Valley of Costa Rica, an area once renowned for its coffee production, which has now largely been converted to residential land due to population growth within the metropolitan area (Jha et al. 2014). A study by Filho et al. (2008) found that the area under coffee in Costa Rica declined 20% from 2001 to 2008, in large part due to urbanization and conversion to pasture. Urbanization has also accounted for 90% of the loss of coffee fields in the western region of El Salvador between 1990 and 2000, and 68% of the clearing in the central region (Blackman et al. 2007). The loss of coffee areas to residential and urban areas has also been reported in the traditional coffee triangle area in Colombia (e.g., Manizales, Pereira; Portafolio 2018; Muñoz-Rios et al. 2020), the Bosquete and Chiriqui regions of Panama (Jha et al. 2014), Guatemala (Jha et al. 2014), and parts of Mexico (Hausermann 2014), but is likely also occurring in other coffee-growing regions near urban centers.

A seventh and final trend that is affecting coffee landscapes is the increase in the coffee area that is being managed under voluntary sustainability standards (VSS), which has the potential to affect the presence of shade trees and forest cover within coffee farms and landscapes. The global area of coffee grown under voluntary sustainability standards — which include both independent, third party certifications (such as Fairtrade, Organic, Rainforest Alliance, 4 C, and UTZ) and private industry standards (such as Starbuck’s C.A.F.E practices and Nestle’s Nespresso AAA programs) — has grown significantly in recent years, increasing 78% from 2011 to 2016 (Lernoud et al. 2018). Latin America (including Brazil) currently provides the majority of the world’s certified coffee, accounting for 72% of the 4C-certified coffee area (in 2016), 67% of the UTZ-certified area (2016), 46% of the organic certified area (2016), 64% of the Rainforest Alliance-certified areas (2016), and 55% of the Fair-trade-certified coffee area (2015; Lernoud et al. 2018). Market trends indicate that the area under VSS-compliant coffee in the region is continuing to increase (Lernoud et al. 2018; Meier et al. 2020), though uptake varies by country and within different regions of individual countries (Lernoud et al. 2018; Grabs et al. 2016).

The growth of VSS-compliant coffee has the potential to increase shade tree diversity, shade density, the extent of forest cover, and landscape connectivity within coffee landscapes, if standards have strong environmental requirements, are applied in a rigorous and transparent way, and result in tangible changes in on-farm tree and forest management (Milder et al. 2014; Elliott 2018). Most of the sustainability standards include measures intended to improve the overall sustainability of coffee production, promote biodiversity conservation, and minimize environmental impacts, in addition to measures to promote social and economic sustainability (Milder et al. 2014). Ecological principles, criteria, and indicators vary among sustainability standards, but can include the use of dense and diverse shade trees, promotion of native trees for shade, protection or restoration of forest areas within coffee farms, maintenance of vegetated riparian buffers, maintenance or restoration of natural ecosystem connectivity, responsible use of agrochemicals, and adoption of good agricultural practices such as soil conservation, among others (Milder et al. 2014; Tscharntke et al. 2015; Bray and Neilson 2017). While the rapid and widespread adoption of certified coffee production by farmers has the potential to reconfigure coffee landscapes and transform the ecological, social, and economic sustainability of coffee production, there are only a handful of studies that have examined the impacts of certification on the structure and composition of coffee landscapes in a rigorous way (Blackman and Rivera 2010; Traldi 2021). One example is a study by Rueda et al. (2015) that found that Rainforest Alliance-certified farms in the Santander region of Colombia had greater tree cover and more diverse tree cover within their coffee plots than non-certified farms, enhancing the overall connectivity of tree and forest cover in the landscape. In addition, certified farmers were more likely to have planted trees outside the coffee plot and to have protected water sources through reforestation (Rueda and Lambin 2013b). In Costa Rica, Guatemala, and Nicaragua, organic coffee farmers had higher shade levels, a greater number of tree species, and more tree strata than conventional farms (Haggar et al. 2015). Other studies show mixed impacts of certification on the composition of coffee farms and landscapes. Blackman and Naranjo (2012), for example, found the organic coffee production by Costa Rican farmers reduced the use of chemical pesticides, fertilizer, and herbicides, but had no significant impact on the use of shade trees or windbreaks. Similarly, Haggar et al. (2017) found variable impacts of certification on the shade tree diversity, species richness, number of tree strata, and density in Nicaragua coffee farms, with some aspects being better on certified farms and others showing no impact of certification. Overall, the extent to which certification impacts the biophysical structure and composition of coffee landscapes is still unclear and merits more vigorous investigation (Bray and Neilson 2017), given the widespread (and growing) area of coffee produced under voluntary sustainability standards.

## Potential ecological, social, and economic consequences of ongoing landscape changes

The rapid and pervasive biophysical changes in coffee farms and landscapes are likely to have significant social, economic, and ecological impacts across the region, both positive and negative (Table 3). However, in most cases, there is insufficient information on what these impacts are or how these impacts may vary in different landscape contexts with different biophysical and socioeconomic characteristics. Here we briefly highlight some of the potential impacts of major transformations in coffee-growing landscapes which require greater attention and research.

**Table 3:** A summary of the patterns, potential drivers, potential social and ecological impacts, and mediating factors of land change in coffee-growing regions of northern and Andean Latin America. The specific relationships between different land-use changes, drivers, impacts, and mediating factors are not yet known and require additional research.

Major transformations of coffee-growing regions	1. Replacement of traditional Arabica coffee varieties with high-yielding, resistant varieties 2. Conventional intensification of coffee production, including the reduction, simplification, or elimination of shade, increased planting densities and increased use of agrochemicals 3. Abandonment of coffee fields and/or conversion of coffee to other agricultural land uses 4. Expansion of coffee production into forested areas, leading to deforestation 5. Introduction and expansion of Robusta coffee 6. Urbanization of coffee-growing regions 7. Increased area of coffee produced under voluntary sustainability standards
Potential drivers of landscape change	Economic drivers: low and volatile coffee prices, high input prices, high labor costs, global market demand and supply, economic impacts of COVID-19 Biophysical drivers: climate change, extreme weather events, coffee leaf rust, other pest, and disease outbreaks Social drivers: aging of coffee farmers, changing importance of coffee within farmer livelihood strategies, shortages of labor, migration, rural conflict Policy drivers: certification processes, growing demand for VSS- compliant coffee, increased demand for specialty coffee, government programs and policies for renovation of coffee plantations, subsidies and fertilizers, private investment, certification processes, sustainability initiatives
Potential impacts of landscape changes	Ecological impacts: biodiversity loss, changes in tree and forest cover on agricultural land, changes in deforestation patterns and associated GHG emissions, soil erosion, changes in forest extent, structure and connectivity, changes in farm and landscape carbon stocks, changes in GHG emissions (from coffee production and deforestation), impacts on ecosystem services (e.g., water, pollination, pest regulation, slope stabilization), contamination of water and soil by fungicides, pesticides, and synthetic fertilizers, changes in the incidence and type of pests and diseases Socioeconomic impacts: changes in farmer food security and nutrition, changes in household income and poverty levels, changes in coffee yield and quality, shifts in livelihood strategies, changes in the relative importance of coffee to farmer livelihoods, impacts on provision of fruits, firewood, timber, and other products for household use and sale, more families engaging in non-farm work, rural conflict (due to alternative, illegal land uses), rural abandonment, increased migration of farmers and coffee laborers to urban areas or other countries, sales of farmland, changes in the adaptive capacity of coffee farmers
Mediating factors	Farm size, land tenure and property rights, proximity to roads and markets, slope and elevation, quality of the coffee-production region, coffee management system, land availability, farmer experience and education, membership in coffee cooperatives, remittances, participation in certification schemes, technical assistance, government and non-governmental programs to support farmers, cultural factors, farm diversification, private sector investment

Of the seven major changes identified above, the intensification of coffee plantations (with less shade, more densely planted coffee bushes, and greater agrochemical use) is the landscape change that has been most closely examined in terms of its potential social, economic, and ecological benefits and risks (e.g., Perfecto et al. 1996, 2019; Rice 1999; Jha et al. 2014). Coffee intensification has significantly increased crop yields and coffee productivity per hectare within coffee farms (Guhl 2008). In some regions, the intensification of coffee production has also enabled farmers to decrease the overall area planted with coffee (as they can produce more coffee on less land), making land available for new agricultural crops and diversifying the composition of coffee landscapes (Guhl 2008). However, the overall impact of intensification on farmer income, livelihoods, and well-being is uncertain due to the high demand for labor and inputs (which are costly), the need to more frequently renovate coffee plantations due to the shorter lifespan of coffee bushes under intensive management, and the homogenization of coffee farming systems and landscapes which makes farmers more vulnerable to soil degradation and climatological or ecological shocks (Rice 1999; Jha et al. 2014; Perfecto et al. 2019). Reductions in the diversity and density of shade trees (especially fruit trees) may also exacerbate food insecurity among smallholder farmers, who harvest fruits to supplement their diets and use on-farm trees as firewood for cooking (Rice 2008; Anderzén et al. 2020). The simplification and loss of shade cover within intensified systems also significantly reduce the value of coffee landscapes for biodiversity conservation and for ecosystem service provision by reducing habitat and resource availability and disrupting landscape connectivity (Perfecto et al. 1996; Moguel and Toledo 1999; Somarriba et al. 2004).

The ongoing expansion of coffee produced under voluntary sustainability standards clearly has the potential to have significant ecological, social, and economic impacts on coffee farms and livelihoods across northern and Andean Latin America, as voluntary standards programs are intentionally designed and implemented to promote socioeconomic and ecological sustainability. Voluntary sustainability standards can enhance the sustainability of coffee production by promoting the adoption of good agricultural practices (including shade management and more responsible use of agrochemicals); improving coffee productivity and marketing; enhancing farmer income, health, and livelihoods; promoting sustainable water use; and avoiding or minimizing negative environmental impacts such as deforestation or forest degradation, among other aspects (Bray and Neilson 2017; Traldi 2021). However, despite the large and rapidly expanding literature on the impacts of voluntary sustainability standards, in particular third-party certification (e.g., Bacon et al. 2008; Blackman and Naranjo 2012; Haggar et al. 2015, 2017), the evidence on ecological, social, and economic outcomes of certification is inconclusive, and certification outcomes often appear to be specific to the contextual and institutional setting or specific certification program implemented (DeFries et al. 2017; Traldi 2021). A review of the impacts of coffee certification programs on smallholder livelihoods, for example, found that while some studies clearly enhance livelihood assets among certified coffee farmers, many studies found either neutral or mixed impacts and a small number even reported negative outcomes (Bray and Neilson 2017). Similarly, a review of the sustainability outcomes of certification of coffee and other tropical agricultural commodities found 34% of the response variables were significantly positive, 58% not significant, and 8% significantly negative (DeFries et al. 2017). Clearly there is a need for more studies on how VSS-compliant coffee affects the structure and composition of coffee landscapes, and influences the economic, social, and economic sustainability of coffee farms and landscapes, using more robust scientific methods that account for self-selection bias (i.e., producers already meeting environmental certification criteria tend to disproportionately obtain certification, Bray and Neilson 2017), include reliable baseline data (to allow for the comparison of conditions before and after certification), and include realistic control groups (Ibanez and Blackman 2016; Bray and Neilson 2017).

The impacts of the ongoing conversion of hundreds of thousands of hectares of coffee plantations with high-yielding resistant varieties are also unknown. The rapid shift to varieties that are resistant to coffee leaf rust has been critical for recovering coffee production across the region, has greatly reduced the risk of harvest loss, and has enabled coffee farmers and laborers to maintain their livelihoods despite the continued presence of coffee leaf rust (Avelino and Anzueto 2020). Other benefits of the resistant varieties include significant increases in coffee production and savings in fungicide use (Avelino and Anzueto 2020). However, since the new varieties are often (but not always) established with little or no shade and require increased use of fertilizers, their adoption could result in a loss of tree cover and the increased contamination of water, with potential negative outcomes for biodiversity and ecosystem services (Jha et al. 2011, 2014; Amico et al. 2020). However, studies on the long-term environmental and social impacts of these changes across the region are not yet available.

The amount of natural forest that is being lost to coffee expansion is not known. Considering that coffee is grown in some of the world’s most important biodiversity hotspots, the clearing of additional forest for coffee production will undoubtedly threaten biodiversity and disrupt the provision of key ecosystem services (Perfecto et al. 1996; Jha et al. 2011). Coffee-driven deforestation will also result in the release of significant amounts of carbon dioxide into the atmosphere, contributing to climate change (Miles and Kapos 2008).

Impacts of the recent introduction of Robusta coffee to new areas in Nicaragua, Mexico, and other regions are equally unclear. On the one hand, Robusta could provide a critical livelihood opportunity for smallholder farmers who live in areas which are currently unsuitable for Arabica cultivation or will become unsuitable for Arabica in the future (Bunn et al. 2015; Nicholson and Menchu 2018). Climate models suggest that the suitable areas for Arabica production in Latin America may be reduced 73–88% by 2050 across different climate scenarios unless adaptation measures are quickly put in place (Imbach et al. 2017), so Arabica coffee may be replaced with Robusta in certain areas in the future. If Robusta coffee replaces subsistence crops (as is happening in parts of Nicaragua, Bjørge 2017), it may impact household food security strategies and potentially affect farmer resilience to climate change and other shocks. The potential impacts of Robusta production on biodiversity conservation, ecosystem service provision, and carbon stocks will depend largely on what land use it replaces and how the Robusta systems are managed. Although Robusta is often cultivated with little or no shade, a recent meta-analysis of the impacts of shade on Robusta cultivation found that shade trees can positively impact growth and yields of Robusta coffee plants, but that the effects of shade vary based on the type of clone planted and the plant age (Piato et al. 2020).

Finally, in areas where coffee is being converted to other agricultural land uses (e.g., pastures, sugarcane, vegetable production, cocoa, citrus) or urban areas, the impacts of these changes will depend on what specific land use replaces coffee and how this land use affects the overall structure, composition, and function (e.g., hydrological) of the landscape. The specific land-use transitions can affect the potential economic profitability of the crop, farmer income levels, labor and input requirements, and whether or not families can continue to make a living from their farm or whether they need to shift to other livelihood strategies, including migration and off-farm work (Hausermann 2014; Bielecki and Wingenbach 2019). If coffee is converted to illegal crops, this shift can also potentially lead to social conflict, as has occurred in certain areas of Colombia where the conversion of coffee to coca production has led to increased violence (Rettberg 2010). The specific type of land-use conversion will also determine changes in on-farm tree cover, in landscape carbon stocks, GHG emissions, pesticide use, and agrochemical contamination of water and soils (Haggar et al. 2013). More detailed information on the specific land-use transitions — and whether these changes are permanent or reversible — is needed to better understand these impacts.

## A research agenda for understanding the drivers, patterns, and potential outcomes of land-use change and informing coffee sustainability policies and practice

Our review shows that coffee-growing regions across northern and Andean Latin America are undergoing rapid and profound transformations, with hundreds of thousands of hectares undergoing changes in coffee varieties, management of shade and inputs, and/or land use. However, information on how, where, and why these changes are occurring, and what the consequences of these changes will be, is extremely scattered and incomplete. Without a more detailed understanding of the rate, magnitude, distribution, and consequences of biophysical changes in coffee landscapes, it is difficult to know which of these landscape trajectories are beneficial for rural livelihoods, biodiversity conservation, climate change mitigation and adaptation, and sustainable development more broadly, and what types of policies, programs, or investments may be needed to promote sustainable coffee landscapes. Here we propose five priority research areas that could greatly enhance our understanding of the dynamics and consequences of changing coffee landscapes in the region and help inform both public and private-sector efforts to enhance coffee sustainability.

First, there is a need to better understand the extent, distribution, rate, and direction of changes in coffee farms and landscapes and to identify the hotspots where rapid and extensive landscape change is occurring and where interventions may be needed. More comprehensive data is needed on both the current distribution, structure, and composition of coffee farms and landscapes (so that there is a consistent baseline from which to measure change) and on the types, rates, and magnitude of landscape change. In particular, more robust information is needed on where coffee production is expanding or contracting, which land uses are replacing coffee (or are being replaced by coffee), where shade cover in coffee plantations is being removed or simplified, where compliance with voluntary sustainability standards are changing on-farm tree and forest cover, where Robusta is expanding, where coffee production is leading to deforestation, and how the structure and composition of coffee-growing farms and landscapes are changing (Blackman et al. 2012; Haggar et al. 2013; Schmitt-Harsh 2013; Guhl 2008; Panhuysen and Pierrot 2018). In addition, it will be important to identify which of these changes are permanent and irreversible (e.g., deforestation to establish coffee plots, or the urbanization of coffee areas), how quickly coffee landscapes are being transformed, and whether there are time lags in responses to particular shocks (e.g., coffee leaf rust, falling prices, COVID-19 impacts, long droughts), so that it is easier to anticipate potential landscape trajectories and their ecological and socioeconomic consequences.

A second research need is to better understand the factors that drive land-use dynamics in coffee landscapes and how these factors interact in different socioecological contexts (Jha et al. 2014). For example, what factors (or combination of factors) spur the conversion of coffee plantations to other land uses, drive the expansion of coffee into new areas, or lead to the replacement of diverse coffee agroforestry systems by open-sun coffee? Or conversely, what factors lead farmers to shift from conventional intensified production to diversified shade-grown production? What factors cause some coffee landscapes to have highly dynamic land use, while others remain relatively stable even amidst rapidly changing contexts (e.g., Hausermann 2014)? While low coffee prices, changing climatic conditions, rising production costs, and coffee leaf rust are clearly driving many of the observed changes in coffee farms and landscapes, there are many additional factors that can transform coffee landscapes, including social factors (e.g., aging of coffee farmers, migration, limited labor availability, social conflict), biophysical factors (e.g., altitude, climate factors, land degradation), economic factors (global coffee demand, certification processes, specialty markets), public policies and institutions (e.g., coffee renovation programs, coca substitution programs, technical assistance, support for ecosystem services), and private investment by the coffee sector (Table 3). The impacts of voluntary sustainability standards merit particular attention given the large and growing areas under third-party certification and private standards, and their potential to significantly impact shade, land use, forest cover, and coffee management. More information is also needed on the potential impacts of the rise in specialty coffee production. It is also important to explore how each of the drivers affects farmer land-use decisions either individually or in combination, and in different socioeconomic and ecological contexts (Bacon et al. 2017). While many of the key drivers are likely specifically related to the coffee sector, there may also be broader factors unrelated to the coffee landscapes to consider (e.g., demographics, urbanization, the COVID-19 pandemic).

A third critical research area is to better understand the socioeconomic and ecological impacts of the changes in coffee landscapes, including potential impacts on coffee production, farmer livelihoods, sustainable development, and the conservation of biodiversity, soil, and water. Coffee farmers across the region are already facing multiple socioeconomic challenges including low-income generation, high poverty levels, recurring food insecurity, high production costs, limited access to education and health services, migration of young male farmers, social conflict, an aging farmer population, and low adaptive capacity (Morris et al. 2013; Baca et al. 2014; Bacon et al. 2017; Harvey et al. 2018; Panhuysen and Pierrot 2018), as well as environmental challenges such as the contamination of water by agrochemicals, soil erosion, biodiversity loss, climate change, and deforestation (Jha et al. 2014; Panhuysen and Pierrot 2018). As discussed in the previous section, it is not yet clear how the ongoing biophysical changes in coffee landscapes will affect landscape-level social, economic, and ecological outcomes. A better understanding of the types, magnitude, and distribution of both positive and negative impacts of different landscape changes — as well as the potential tradeoffs and synergies across different social, economic, and environmental outcomes — is critical for informing ongoing policies, programs, initiatives, and financial incentives designed to enhance the overall sustainability of the coffee sector.

A fourth research need is to explore the mediating factors that explain why the same drivers or set of drivers sometimes lead to very different outcomes in different landscapes. For example, why have price shocks in some places led to the abandonment of diverse, rustic coffee while in other regions, these systems have persisted despite (or perhaps because of) shocks (Hausermann 2014)? Why has the adoption of new rust-resistant varieties led to the elimination of shade in some areas but not in others? Many potential factors may mediate the impacts of different drivers or shocks within a given landscape (Bosselmann 2012), such as farm characteristics or management (e.g., topography, farm size, elevation, coffee varieties, coffee productivity), farmer and household characteristics (e.g., family size, dependence on coffee production, education levels, market access, off-farm labor opportunities, migration), and institutional aspects (e.g., technical support, policies, coffee cooperatives). However, we have little understanding of how different factors (or combinations of factors) affect both the trajectory of change and overall impacts on farmer livelihoods, rural economies, and the environment within individual landscapes.

A final research area is the development of innovative modeling and analytical methods for the study of these complex socio-ecological systems, including farm system modeling, landscape modeling, participatory scenario modeling, agent-based modeling, and other approaches (e.g., Parker et al. 2003; Matthews et al. 2007; Speelman et al. 2014; Johnson and Karlberg 2017; Meijer et al. 2018; Rahn et al. 2018). These models should (1) allow the exploration of different scenarios of changes, for instance, in the extent, distribution, and management of Arabica and/or Robusta coffee, different shade systems, and other land uses within coffee-growing landscapes; (2) analyze the potential socioeconomic and ecological outcomes of different farming systems design and landscape trajectories for different stakeholder groups; (3) identify potential tradeoffs and synergies across different production and landscape goals (e.g., improved farmer livelihoods, coffee production, climate change mitigation, ecosystem service provision, biodiversity conservation); (4) take into account both the drivers and mitigating factors affecting landscape change; and (5) examine how different interventions (e.g., policy interventions, market prices, certification premiums, payments for ecosystem services) are likely to influence the trajectory of a particular coffee-growing landscape and affect its social, ecological, and economic sustainability. Such scenario-building and modeling efforts will be critical for helping coffee institutes, public entities, research organizations, agricultural extension services, private companies, the financial sector, and other stakeholders identify which types of interventions are likely to lead to sustainable landscape trajectories that deliver the desired environmental and socioeconomic goals, and can be used to inform the design of effective system designs, policies, and incentives.

Moving this ambitious research agenda forward will require greatly improving the availability and quality of biophysical and socioeconomic data on the region’s coffee farms, landscapes, and farmers and developing new research and development collaborations. There is an urgent need for comprehensive, spatially explicit, and up-to-date information on where and how coffee is grown in each country, with details on farm size, coffee area, coffee management (e.g., planting density, shade type, agrochemical use, Haggar et al. 2013), certification status, coffee varieties and species, coffee age, yield, and the composition and structure of coffee landscapes (including information on other crops, pastures, forests, and residential areas), so that there are clear baselines against which future landscape changes can be measured. It is critical that biophysical data on coffee landscapes is linked to key socioeconomic data regarding coffee farmers and their livelihoods (e.g., family size, farmer age, level of education, income levels, participation in certification schemes, participation in farmer cooperatives, labor use, other crops they cultivate, non-farming sources of income, the relative importance of coffee relative to other income sources). This linking allows the exploration of interactions and feedbacks among socioeconomic and ecological aspects. In order for these data to be useful for decision-making, datasets should be regularly updated using consistent methodologies so that it is possible to quickly detect changes in both biophysical and socioeconomic conditions and take the necessary actions to address any trends that threaten sustainability of coffee landscapes and livelihoods.

Some of this spatially explicit data is already available from national coffee institutes, national government censuses, or the private sector (which typically have their own proprietary information on the landscapes where they source coffee from), but increased cooperation and data sharing are necessary. In the many coffee-growing landscapes where data is missing, incomplete, or out-of-date, national-level surveys or censuses by governments, national coffee institutes, or agricultural extension agencies could be adjusted to provide the required data on household, farm, and landscape characteristics and dynamics. Recent advances in remote sensing and machine learning could also make it easier in the future to track coffee production; identify different land uses within coffee landscapes; monitor changes in landscape structure, composition, and function; and potentially also characterize shade levels within coffee fields (Hunt et al. 2020). Long-term research on coffee landscape structure, patterns, and socioecological processes could be centered on a network of coffee “sentinel landscapes” representing a minimum, but sufficient, set of socioecological contexts in which a broad range of biophysical, social, and economic characteristics and processes are monitored using a consistent set of methods (Dewi et al. 2017). Such research networks would ideally be supported by a broad suite of stakeholders (including farmers, agricultural research centers, academia, public sector organizations, NGO’s, academia, coffee cooperatives, coffee retailers, and other private sector entities), all of whom stand to benefit from more sustainable coffee landscapes.

## Conclusions

Coffee farms and landscapes across northern and Andean Latin America are undergoing rapid and profound biophysical changes in response to low coffee prices, coffee leaf rust, changing climatic conditions, and other factors. Key changes include the widespread adoption of new rust-resistant varieties, the conventional intensification of coffee production, the abandonment and conversion of coffee to other land uses, the expansion of coffee into forested areas, the introduction of Robusta coffee, the urbanization of coffee regions, and the increase in the area of coffee produced under voluntary sustainability standards. All of these changes have the potential to profoundly change the sustainability of the coffee sector in different ways (both positive and negative), yet, as our review demonstrates, there is insufficient and scattered information on how, where, and why different land-use and landscape changes are occurring, and the ways in which they will affect coffee production, farmer livelihoods, ecosystem services, and other aspects of sustainable development. The research agenda we have laid out aims to address these knowledge gaps and ensure that governments, the private sector, NGO’s, agricultural technicians, and other stakeholders have the information to make strategic and informed decisions about their efforts to promote sustainable coffee systems and landscapes across the region. It can also inform ongoing sustainability efforts in other coffee-growing regions across the tropics which are undergoing some of the same landscape transformations and grappling with similar sustainable development and conservation challenges (e.g., Garcia et al. 2010; Hylander et al. 2013; Millard 2017). While our research agenda is ambitious and will require significant investment, it is critical for better understanding both the current and future contexts of coffee landscapes and communities, and their contribution to sustainable development. A better understanding of the drivers, patterns, and outcomes of ongoing landscape change is vital for steering the global coffee sector towards landscape trajectories that lead to desirable socioeconomic and environmental outcomes.

## Supplementary Information

ESM 1 (DOCX 22 kb)

## References

[CR1] Aceves Navarro LA, Rivera Hernández B, López Castañeda A, Palma López DJ, González Mancillas R, Juárez López JF (2018). Áreas potenciales y vulnerabilidad del cultivo de café tipo robusta (*Coffea canephora* P.) al cambio climático en el estado de Tabasco, México. Nova Scientia.

[CR2] Amico A, Paz-Pellat F (2018). Del papel a la acción en la mitigación y adaptación al cambio climático: la roya del cafeto en Chiapas. Madera y bosques.

[CR3] Amico AM, Ituarte-Lima C, Elmqvist T (2020). Learning from social–ecological crisis for legal resilience building: multi-scale dynamics in the coffee rust epidemic. Sustain Sci.

[CR4] Anacafé (2019) Café de Guatemala en cifras: datos actividad cafetalera nacional 2018-2019. https://www.anacafe.org/uploads/file/cd2552c54b3a4616b0e82ae14c7db79a/GuatemalaCafeenCifras-2018-2019.pdf. Accessed 20 Oct 2020

[CR5] Anderzén J, Luna AG, Luna-González DV, Merrill SC, Caswell M, Méndez VE, Jonapá RH (2020). Effects of on-farm diversification strategies on smallholder coffee farmer food security and income sufficiency in Chiapas, Mexico. J of Rural Stud.

[CR6] Aquino M (2020) Producción peruana de café cae 10% por abandono de cultivos y menos mano de obra. Reuters. https://www.reuters.com/article/cafe-peru-idLTAKBN26S34R. Accessed 2 Apr 2021

[CR7] Avelino J, Anzueto F (2020) Coffee Leaf Rust epidemics in Central America: chronicle of a resistance breakdown following the great epidemics of 2012 and 2013. In: Emerging Plant Diseases and Global Food Security. The American Phytopathological Society, pp. 185–198. 10.1094/9780890546383.009

[CR8] Avelino J, Cristancho M, Georgiou S, Imbach P, Aguilar L, Bornemann G, Läderach P, Anzueto F, Hruska AJ, Morales C (2015). The coffee rust crises in Colombia and Central America (2008–2013): impacts, plausible causes and proposed solutions. Food Secur.

[CR9] Babin N (2015). The coffee crisis, fair trade, and agroecological transformation: impacts on land-use change in Costa Rica. Agroecol Sust Food.

[CR10] Babin N (2020). Class differentiation, deagrarianization, and repeasantization following the coffee crisis in Agua Buena, Costa Rica. J Agrar Chang.

[CR11] Baca M, Läderach P, Haggar J, Schroth G, Ovalle O (2014). An integrated framework for assessing vulnerability to climate change and developing adaptation strategies for coffee growing families in Mesoamerica. PLoS One.

[CR12] Bacon CM, Ernesto Mendez V, Gómez MEF, Stuart D, Flores SRD (2008). Are sustainable coffee certifications enough to secure farmer livelihoods? The Millenium Development Goals and Nicaragua’s Fair-Trade cooperatives. Globalizations.

[CR13] Bacon CM, Sundstrom WA, Stewart IT, Beezer D (2017). Vulnerability to cumulative hazards: coping with the coffee leaf rust outbreak, drought, and food insecurity in Nicaragua. World Dev.

[CR14] Bager SL, Lambin EF (2020). Sustainability strategies by companies in the global coffee sector. Bus Strateg Environ.

[CR15] Banco Central de Reserva del Peru (2020) Cuadros estadísticos. Lima, Peru. https://www.bcrp.gob.pe/estadisticas/cuadros-de-la-nota-semanal.html Accessed 7 Oct 2020

[CR16] Barrera JF (2016) Café robusta, ¿héroe o villano? Ecofronteras 20:14-17. https://revistas.ecosur.mx/ecofronteras/index.php/eco/article/view/1661 Accessed 20 Oct 2020

[CR17] Baumeister E (2017). Transición cafetalera en América Central: de haciendas hacia una mayor presencia productiva de pequeños y medianos productores. Revista Interdisciplinaria de Estudios Agrarios.

[CR18] Bielecki CD, Wingenbach G (2019). Using a livelihoods framework to analyze farmer identity and decision making during the Central American coffee leaf rust outbreak: implications for addressing climate change and crop diversification. Agroecol Sust Food.

[CR19] Bjørge HH (2017) The expansion of coffee Robusta (*C. canephora*) in Nicaragua. Master thesis. Norwegian University of Life Sciences. https://nmbu.brage.unit.no/nmbu-xmlui/handle/11250/2449175. Accessed 31 Oct 2020

[CR20] Blackman A, Naranjo MA (2012). Does eco-certification have environmental benefits? Organic coffee in Costa Rica. Ecol Econ.

[CR21] Blackman A, Rivera JE (2010) The evidence base for environmental and socioeconomic impacts of ‘sustainable’ certification. Resources for the Future. Washington D.C. https://media.rff.org/documents/RFF-DP-10-17.pdf. Accessed 10 Apr 2021

[CR22] Blackman A, Albers HJ, Avalos-Sartorio B, Crooks L (2005) Deforestation and shade coffee in Oaxaca, Mexico: key research findings. Discussion paper 05-39. Resources for the Future, Washington D.C. 10.22004/AG.ECON.10799

[CR23] Blackman A, Ávalos-Sartorio B, Chow J (2007). Shade coffee & tree cover loss: lessons from El Salvador. Environ Sci Policy Sustain Dev.

[CR24] Blackman A, Ávalos-Sartorio B, Chow J (2012). Land cover change in agroforestry: shade coffee in El Salvador. Land Econ.

[CR25] Bosselmann AS (2012). Mediating factors of land use change among coffee farmers in a biological corridor. Ecol Econ.

[CR26] Bray JG, Neilson J (2017). Reviewing the impacts of coffee certification programmes on smallholder livelihoods. Int J Biodivers Sci Ecosyst Serv Manag.

[CR27] Bunn C, Läderach P, Ovalle Rivera O, Kirschke D (2015). A bitter cup: climate change profile of global production of Arabica and Robusta coffee. Clim Chang.

[CR28] Bunn C, Lundy M, Läderach P, Girvetz E, Castro F (2018) Climate Smart coffee in Honduras. International Center for Tropical Agriculture (CIAT), United States Agency for International Development (USAID). Cali. CO. 27 p. https://hdl.handle.net/10568/97530 Accessed 23 Oct 2020

[CR29] Bunn C, Lundy M, Läderach P, Castro-Llanos F, Fernandez-Kolb P, Rigsby D (2019) Climate-smart Coffee in Guatemala. International Center for Tropical Agriculture (CIAT), Cali, CO. 28 p. https://hdl.handle.net/10568/103771 Accessed 23 Oct 2020

[CR30] CABI (2018) El estado actual de la rentabilidad del café en Centroamérica. PROMECAFE. Guatemala. https://promecafe.net/?p=5405 Accessed 20 Oct 2020

[CR31] Canet Brenes G, Soto Víquez C, Ocampo Thomason P, Rivera Ramírez J, Navarro Hurtado A, Guatemala Morales G, Villanueva Rodríguez S (2016) La situación y tendencias de la producción de café en América Latina y El Caribe. Instituto Interamericano de Cooperación para la Agricultura y Centro de Investigación y Asistencia en Tecnología y Diseño del Estado de Jalisco. San José, Costa Rica. http://repositorio.iica.int/handle/11324/2792 Accessed 20 Oct 2020

[CR32] Cerda R, Allinne C, Gary C, Tixier P, Harvey CA, Krolczyk L, Mathiot C, Clément E, Aubertot JN, Avelino J (2017). Effects of shade, altitude and management on multiple ecosystem services in coffee agroecosystems. Eur J Agron.

[CR33] Consejo Salvadoreño del Café (2020) Estadísticas Cafetaleras al 31 de Julio de 2020 Informe Oficial. Gobierno de El Salvador. La Libertad, El Salvador. http://laip.csc.gob.sv/marco-de-gestion-estrategica/estadisticas/ Accessed 20 Oct 2020

[CR34] Cortina-Villar S, Plascencia-Vargas H, Vaca R, Schroth G, Zepeda Y, Soto-Pinto L, Nahed-Toral J (2012). Resolving the conflict between ecosystem protection and land use in protected areas of the Sierra Madre de Chiapas, Mexico. Environ Manag.

[CR35] Covaleda S, Aguilar S, Ranero A, Marín I, Paz F (2014) Diagnóstico sobre determinantes de deforestación en Chiapas, México. Alianza México para la reducción de emisiones por deforestación y degradación (Alianza México-REDD+), Agencia de los Estados Unidos para el Desarrollo Internacional (USAID). http://sis.cnf.gob.mx/wp-content/plugins/conaforfiles/2018/nacional/catalogo/biblioteca/101.pdf Accessed 20 Oct 2020

[CR36] DeFries RS, Fanzo J, Mondal P, Remans R, Wood SA (2017). Is voluntary certification of tropical agricultural commodities achieving sustainability goals for small-scale producers? A review of the evidence. Environ Res Lett.

[CR37] Dewi S, Van Noordwijk M, Zulkarnain MT, Dwiputra A, Hyman G, Prabhu R, Gitz V, Nasi R (2017). Tropical forest-transition landscapes: a portfolio for studying people, tree crops and agro-ecological change in context. Int J Biodivers Sci Ecosyst Serv Manag.

[CR38] Dupre SI (2018) Coffee leaf rust and smallholder farmers in Guatemala: livelihood impacts, migration and other coping strategies. Doctoral Dissertation, University of Maryland Baltimore County, USA

[CR39] Eakin HC, Webbe MB (2009). Linking local vulnerability to system sustainability in a resilience framework: two cases from Latin America. Clim Chang.

[CR40] Eakin H, Tucker C, Castellanos E, Díaz R, Barrera JF, Morales H (2014). Adaptation in a multi-stressor environment: perceptions and responses to climatic and economic risks by coffee growers in Mesoamerica. Environ Dev Sustain.

[CR41] Elliott KA (2018) What are we getting from voluntary sustainability standards for coffee? Center for Global Development, Policy Paper 129. https://cisp.cachefly.net/assets/articles/attachments/75587_what-are-we-getting-voluntary-sustainability-standards-coffee.pdf Accessed 10 Apr 2021

[CR42] Ellis EA, Baerenklau KA, Marcos-Martínez R, Chávez E (2010). Land use/land cover change dynamics and drivers in a low-grade marginal coffee growing region of Veracruz, Mexico. Agrofor Syst.

[CR43] Escobedo Aguilar A, Bendaña E, Gutierrez R (2017) Cartilla Cadena de Valor Café de Nicaragua. CATIE, Costa Rica. 10.13140/RG.2.2.17053.15842

[CR44] Favovich M (2020) ¿Que incentiva la industria del café en Panamá? Asamblea Nacional Panamá https://espaciocivico.org/wp-content/uploads/2020/02/que_incentiva_la_industria_del_cafe_en_panama.pdf Accessed 2 Oct 2020

[CR45] Federación Nacional de Cafeteros de Colombia (2019) Estadísticas Cafeteras: precios, área y producción del café. Bogota, Colombia. https://federaciondecafeteros.org/wp/estadisticas-cafeteras/. Accessed 2 Oct 2020

[CR46] Fernandez-Kolb P, Castro-Llanos F, Martínez-Valle A, Siles P, Läderach P, Lundy M, Bunn C (2019) Climate-smart Coffee in El Salvador. International Center for Tropical Agriculture (CIAT), Cali, Colombia. 22 p. https://hdl.handle.net/10568/103773. Accessed 23 Oct 2020

[CR47] Flores M, Bratescu A, Martinez JO, Oviedo JA, Acosta A (2002) Centroamérica: El impacto de la caída de los precios del café. Naciones Unidas, Mexico D.F. ISBN: 92-1-322015-4 https://repositorio.cepal.org/handle/11362/5016 Accessed 25 Oct 2020

[CR48] Virginio Filho, E de Melo, Abarca S (2008) Cafetales para servicios ecosistémicos, con énfasis en el potencial de sumideros de carbono. CATIE-COOCAFE-FUNCAFOR, Costa Rica. https://www.researchgate.net/profile/Elias_De_Melo_Virginio_Filho/publication/272181060_Cafetales_para_servicios_ecosistemicos_con_enfasis_en_el_potencial_de_sumideros_de_carbono_El_caso_de_cooperativas_cafetaleras_afiliadas_a_COOCAFE_Costa_Rica/links/54de79640cf2510fcee3d7cc/Cafetales-para-servicios-ecosistemicos-con-enfasis-en-el-potencial-de-sumideros-de-carbono-El-caso-de-cooperativas-cafetaleras-afiliadas-a-COOCAFE-Costa-Rica.pdf Accessed 25 Oct 2020

[CR49] Ganzenmuller R, Castro Nuñez A (2019) Project report: links between deforestation and cacao, coffee, palm oil and cattle production in Peru. International Center for Tropical Agriculture (CIAT), Cali, Colombia

[CR50] Garcia CA, Bhagwat SA, Ghazoul J, Nath CD, Nanaya KM, Kushalappa CG, Raghuramulu Y, Nasi R, Vaast P (2010). Biodiversity conservation in agricultural landscapes: challenges and opportunities of coffee agroforests in the Western Ghats, India. Conserv Biol.

[CR51] García C, García J, Ochoa G, Mora J, Castellanos J. 2014. Impact Evaluation of UTZ Certified Coffee Program in Colombia. (2008-2012). CRECE. Manizales, Colombia. https://utz.org/wpcontent/uploads/2015/12/UTZ-CRECE-WEB.pdf Accessed 7 Oct 2020

[CR52] Gonzalez D (2016) Gobierno da luz verde a café robusta. Editorial La Prensa, S.A. Managua, Nicaragua. http://www.laprensa.com.ni/2016/12/05/economia/2145864-gobierno-da-luz-verde-acafe-robusta. Accessed 09 Sept 2020

[CR53] Grabs J, Kilian B, Hernández DC, Dietz T (2016). Understanding coffee certification dynamics: a spatial analysis of voluntary sustainability standard proliferation. Int Food Agribusiness Manag Rev.

[CR54] Grisaffi T, Farthing L (2021) Cociane: falling coffee prices force Peru’s farmers to cultivate coca. The Conservation. https://theconversation.com/cocaine-falling-coffee-prices-force-perus-farmers-to-cultivate-coca-154754 Accessed 19 Apr 2021

[CR55] Guhl A (2008) Café y cambio de paisaje en Colombia, 1970-2005. Fondo Editorial Universidad EAFIT, Medellín, Colombia. https://babel.banrepcultural.org/digital/collection/p17054coll18/id/378 Accessed 2 Oct 2020

[CR56] Guido Z, Knudson C, Rhiney K (2020). Will COVID-19 be one shock too many for smallholder coffee livelihoods?. World Dev.

[CR57] Haggar J, Medina B, Aguilar RM, Muñoz C (2013). Land use change on coffee farms in southern Guatemala and its environmental consequences. Environ Manag.

[CR58] Haggar J, Asigbaase M, Bonilla G, Pico J, Quilo A (2015). Tree diversity on sustainably certified and conventional coffee farms in Central America. Biodivers Conserv.

[CR59] Haggar J, Soto G, Casanoves F, de Melo Virginio E (2017). Environmental-economic benefits and trade-offs on sustainably certified coffee farms. Ecol Indic.

[CR60] Harvey CA, Martínez-Rodríguez MR, Cárdenas JM, Avelino J, Rapidel B, Vignola R, Donatti CI, Vílchez-Mendoza S (2017). The use of Ecosystem-based Adaptation practices by smallholder farmers in Central America. Agric Ecosyst Environ.

[CR61] Harvey CA, Saborio-Rodríguez M, Martínez-Rodríguez MR, Viguera B, Chain-Guadarrama A, Vignola R, Alpízar F (2018). Climate change impacts and adaptation among smallholder farmers in Central America. Agric Food Secur.

[CR62] Hausermann H (2014). Maintaining the coffee canopy: understanding change and continuity in Central Veracruz. Hum Ecol.

[CR63] Hite EB, Bray DB, Duran E, Rincón-Gutiérrez A (2017). From forests and fields to coffee and back again: historic transformations of a traditional coffee agroecosystem in Oaxaca, Mexico. Soc Nat Resour.

[CR64] Hunt DA, Tabor K, Hewson JH, Wood MA, Reymondin L, Koenig K, Schmitt-Harsh M, Follett F (2020). Review of remote sensing methods to map coffee production systems. Remote Sens.

[CR65] Hylander K, Nemomissa S, Delrue J, Enkosa W (2013). Effects of coffee management on deforestation rates and forest integrity. Conserv Biol.

[CR66] Ibanez M, Blackman A (2016). Is eco-certification a win–win for developing country agriculture? Organic coffee certification in Colombia. World Dev.

[CR67] ICAFE (2020) Informe sobre la actividad cafetalera de Costa Rica. Instituto del Café de Costa Rica. Heredia, Costa Rica. http://www.icafe.cr/wpcontent/uploads/informacion_mercado/informes_actividad/actual/Informe%20Actividad%20Cafetalera.pdf Accessed 7 Oct 2020

[CR68] ICO (2014) World coffee trade (1963 – 2013): a review of the markets, challenges and opportunities facing the sector. International Coffee Organization, London, UK. http://www.ico.org/news/icc-111-5-r1e-world-coffee-outlook.pdf Accessed 22 Sep 2020

[CR69] ICO (International Coffee Organization) (2019) Coffee development report. Growing for prosperity: economic viability as the catalyst for a sustainable coffee sector. International Coffee Organization, London. https://www.internationalcoffeecouncil.org/media/coffeeDevelopmentReport.pdf Accessed 22 Sept 2020

[CR70] IDH (2019) Coffee production in the face of climate change: country profiles. IDH The Sustainable Trade Initiative. Utrecht, Netherlands. https://www.idhsustainabletrade.com/publication/coffee-production-in-the-face-of-climate-change/ Accessed 20 Oct 2020

[CR71] Imbach P, Fung E, Hannah L, Navarro-Racines CE, Roubik DW, Ricketts TH, Harvey CA, Donatti CI, Läderach P, Locatelli B, Roehrdanz PR (2017). Coupling of pollination services and coffee suitability under climate change. PNAS.

[CR72] Instituto Nacional de Estadística y Censos (INEC) (2019) Encuesta de superficie y producción agropecuaria continua Ecuador. Compendio estadístico. Quito, Ecuador. https://www.ecuadorencifras.gob.ec/encuesta-de-superficie-y-produccion-agropecuaria-continua-bbd/ Accessed 23 Oct 2020

[CR73] ITC (International Trade Center) (2011) Coffee exporter’s guide: third edition. ITC, Geneva, Switzerland. 247 pp. https://www.intracen.org/the-coffee-exporters-guide-third-edition/ Accessed 20 Oct 2020

[CR74] Jezeer RE, Verweij PA, Boot R, Junginger M, Santos MJ (2019). Influence of livelihood assets, experienced shocks and perceived risks on smallholder coffee farming practices in Peru. J Environ Manag.

[CR75] Jha S, Bacon CM, Philpott SM, Rice RA, Méndez VE, Läderach P (2011) A review of ecosystem services, farmer livelihoods, and value chains in shade coffee agroecosystems. In: Integrating agriculture, conservation and ecotourism: examples from the field. Springer Netherlands, Dordrecht, pp 141–208

[CR76] Jha S, Bacon CM, Philpott SM, Méndez VE, Läderach P, Rice RA (2014). Shade coffee: update on a disappearing refuge for biodiversity. Bioscience.

[CR77] Johnson OW, Karlberg L (2017). Co-exploring the water-energy-food nexus: facilitating dialogue through participatory scenario building. Front Env Sci.

[CR78] Läderach P, Ramirez-Villegas J, Navarro-Racines C, Zelaya C, Martinez-Valle A, Jarvis A (2017). Climate change adaptation of coffee production in space and time. Clim Chang.

[CR79] León-Carrasco JA (2020) Destacó el ministro de Relaciones Exteriores, Mario López Chavarri: el café es fuente de empleo para 2 millones de peruanos en toda la cadena agroproductiva. Agencia Agraria de Noticias. Peru. https://agraria.pe/quienes-somos Accessed 07 October 2020

[CR80] Lernoud J, Potts J, Sampson G, Schlatter B, Huppe G, Voora V, Willer H, Wozniak J, Dang D (2018) The state of sustainable markets- statistics and emerging trends. ITC, Geneva. https://www.intracen.org/uploadedFiles/intracenorg/Content/Publications/Sustainibility 2018 layout-FIN-web-v1.pdf Accessed Apr 15, 2021

[CR81] Marquardt K, Pain A, Bartholdson Ö, Rengifo LR (2019). Forest dynamics in the Peruvian Amazon: understanding processes of change. Small-scale For.

[CR82] Matthews RB, Gilbert NG, Roach A, Polhill JG, Gotts NM (2007). Agent-based land-use models: a review of applications. Landsc Ecol.

[CR83] Meier C, Sampson G, Larrea C, Schlatter B, Voora V, Dang D, Bermundez S, Wozniak J, Willer H (2020) The state of sustainable markets 2020: statistics and emerging trends. ITC, Geneva. Available at: https://www.intracen.org/publication/Sustainable-Markets-2020/ Accessed 10 Apr 2021

[CR84] Meijer J, Shames S, Scherr SJ, Giesen P (2018) Spatial modelling of participatory landscape scenarios: synthesis and lessons learned from exploring potential SDG progress in 3 case studies. PBL Netherlands Environmental Assessment Agency. The Hauge, The Netherlands. https://ecoagriculture.org/publication/spatial-modelling-of-participatory-landscape-scenarios/ Accessed 25 Oct 2020

[CR85] Méndez VE, Bacon CM, Olson M, Morris KS, Shattuck A (2010). Agrobiodiversity and shade coffee smallholder livelihoods: a review and synthesis of ten years of research in Central America. Prof Geogr.

[CR86] MIDA (2018) Información General, Año 2017-2018. Ministerio de Desarrollo Agropecuario, República de Panamá. https://mida.gob.pa/upload/documentos/2017-2018cierre(1).pdf Accessed 7 Oct 2020

[CR87] Milder JC, Arbuthnot M, Blackman A, Brooks SE, Giovannucci D, Gross L, Kennedy ET, Komives K, Lambin EF, Lee A, Meyer D (2014). An agenda for assessing and improving conservation impacts of sustainability standards in tropical agriculture. Conserv Biol.

[CR88] Miles L, Kapos V (2008). Reducing greenhouse gas emissions from deforestation and forest degradation: global land-use implications. Science.

[CR89] Millard E (2017). Still brewing: fostering sustainable coffee production. World Dev Perspect.

[CR90] Moguel P, Toledo VM (1999). Biodiversity conservation in traditional coffee systems of Mexico. Conserv Biol.

[CR91] Morris KS, Mendez VE, Olson MB (2013). ‘Los meses flacos’: seasonal food insecurity in a Salvadoran organic coffee cooperative. J Peasant Stud.

[CR92] Muñoz-Rios LA, Vargas-Villegas J, Suarez A (2020). Local perceptions about rural abandonment drivers in the Colombian coffee region: insights from the city of Manizales. Land Use Policy.

[CR93] Nicholson M (2014) Ravaged by roya; Central American farmers abandon coffee for tomatoes. Reuters. https://www.reuters.com/article/coffee-leafrust-el-salvador/ravaged-by-roya-central-american-farmers-abandon-coffee-for-tomatoes-idUSL2N0S21DJ20141010 Accessed 2 Oct 2020

[CR94] Nicholson M, Menchu S (2018) Latin America’s premium coffee growers branch out to cheaper beans. Reuters. https://www.reuters.com/article/us-latam-coffee-robusta/ latin-americas-premium-coffee-growers-branch-out-to-cheaper-beans-idUSKBN1FR0JK Accessed 2 Oct 2020

[CR95] Panhuysen S, Pierrot J (2018) Coffee Barometer 2018. Hivos. https://www.hivos.org/assets/2018/06/Coffee-Barometer-2018.pdf Accessed 2 Oct 2020

[CR96] Panhuysen, S. Pierrot J (2020) Coffee Barometer 2020. Hivos. https://coffeebarometer.org/ Accessed 03 April 2020

[CR97] Parker DC, Manson SM, Janssen MA, Hoffmann MJ, Deadman P (2003). Multi-agent systems for the simulation of land-use and land-cover change: a review. Ann Assoc Am Geogr.

[CR98] Perfecto I, Rice RA, Greenberg R, Van der Voort ME (1996). Shade coffee: a disappearing refuge for biodiversity: shade coffee plantations can contain as much biodiversity as forest habitats. Bioscience.

[CR99] Perfecto I, Jiménez-Soto ME, Vandermeer J (2019). Coffee landscapes shaping the anthropocene: forced simplification on a complex agroecological landscape. Curr Anthropol.

[CR100] Piato K, Lefort F, Subía C, Caicedo C, Calderón D, Pico J, Norgrove L (2020). Effects of shade trees on Robusta coffee growth, yield and quality. A meta-analysis Agron Sustain Dev.

[CR101] Portafolio (2017) El café robusto seduce a los productores colombianos. Portafolio. Bogotá, Colombia. https://www.portafolio.co/negocios/cafe-robusta-seria-sembrado-en-colombia-504327 Accessed 02 Oct 2020

[CR102] Portafolio (2018) Así cambió el mapa cafetero en lo corrido del siglo XX. Portafolio. Bogotá, Colombia. https://www.portafolio.co/economia/asi-cambio-el-mapa-cafetero-en-lo-corrido-del-siglo-xxi-523733 Accessed 2 Oct 2020

[CR103] Pretel EA (2018) Exclusive: Costa Rica to lift 30-year ban on planting robusta coffee trees. Reuters. https://www.reuters.com/article/us-costa-rica-coffee-exclusive-idUSKBN1FT2UH Accessed 02 Oct 2020

[CR104] Quiroga S, Suárez C, Diego Solís J, Martinez-Juárez P (2020). Framing vulnerability and coffee farmers’ behaviour in the context of climate change adaptation in Nicaragua. World Dev.

[CR105] Rahn E, Vaast P, Läderach P, Asten P, Jassogne L, Ghazoul J (2018). Exploring adaptation strategies of coffee production to climate change using a process-based model. Ecol Model.

[CR106] Rettberg A (2010). Global markets, local conflict: violence in the Colombian coffee region after the breakdown of the International Coffee Agreement. Lat Am Perspect.

[CR107] Rice RA (1999). A place unbecoming: the coffee farm of Northern Latin America. Geogr Rev.

[CR108] Rice RA (2008). Agricultural intensification within agroforestry: the case of coffee and wood products. Agric Ecosyst Environ.

[CR109] Romero CA (2020) Observatorio de commodities café 2020. Ministerio de agricultura y riego. Lima, Peru. https://www.inia.gob.pe/wp-content/uploads/2020/04/Reporte_Obs_Commodities_Cafe.pdf Accessed 04 Oct 2020

[CR110] Rueda X, Lambin EF (2013). Linking globalization to local land uses: how eco-consumers and gourmands are changing the Colombian coffee landscapes. World Dev.

[CR111] Rueda X, Lambin EF (2013). Responding to globalization: impacts of certification on Colombian small-scale coffee growers. Ecol Soc.

[CR112] Rueda X, Thomas NE, Lambin EF (2015). Eco-certification and coffee cultivation enhance tree cover and forest connectivity in the Colombian coffee landscapes. Reg Environ Chang.

[CR113] Schlesinger P, Muñoz Brenes CL, Jones KW, Vierling LA (2017). The Trifinio Region: a case study of transboundary forest change in Central America. J Land Use Sci.

[CR114] Schmitt-Harsh M (2013). Landscape change in Guatemala: driving forces of forest and coffee agroforest expansion and contraction from 1990 to 2010. Appl Geogr.

[CR115] Secretaría de Agricultura y Desarrollo Rural (2018) México, onceavo productor mundial de café. Mexico City, Mexico. https://www.gob.mx/agricultura/es/articulos/mexico-onceavo-productor-mundial-de-cafe?idiom=es#:~:text=Actualmente%2C%20el%20caf%C3%A9%20representa%20el,la%20producci%C3%B3n%20de%20bienes%20agroindustriales Accessed Oct 02 2020

[CR116] Semple K (2019) Central American farmers head to the U.S., fleeing climate change. The New York Times, April 13, 2019, New York. https://www.nytimes.com/2019/04/13/world/americas/coffee-climate-change-migration.html Accessed 7 Oct 2020

[CR117] Somarriba E, López Sampson A (2018) Coffee and cocoa agroforestry systems: pathways to deforestation, reforestation, and tree cover change. PROFOR. Background paper. CATIE, Turrialba, Costa Rica. https://www.profor.info/sites/profor.info/files/Coffee_Case%20study_LEAVES_2018.pdf Accessed 25 Oct 2020

[CR118] Somarriba E, Harvey C, Samper M, Anthony F, González JE, Staver C, Rice R, Schroth G, Fonseca GD, Gascon C, Vasconcelos HL, Izac AM (2004) Biodiversity conservation in neotropical coffee (*Coffea arabica*) plantations. In: Agroforestry and Biodiversity Consservation in Tropical Landscapes. Island Press. Washington D.C, United States, pp 198–266

[CR119] Speelman EN, García-Barrios LE, Groot JCJ, Tittonell P (2014). Gaming for smallholder participation in the design of more sustainable agricultural landscapes. Agric Syst.

[CR120] Terazono E, Webber J, Schipani A (2019) The abandoned farms behind the global coffee craze. Financial Times. https://www.ft.com/content/5009be96-7569-11e9-be7d-6d846537acab Accessed 10 Oct 2020

[CR121] Traldi R (2021). Progress and pitfalls: a systematic review of the evidence for agricultural sustainability standards. Ecol Indic.

[CR122] Tscharntke T, Milder JC, Schroth G, Clough Y, DeClerck F, Waldron A, Rice R, Ghazoul J (2015). Conserving biodiversity through certification of tropical agroforestry crops at local and landscape scales. Conserv Lett.

[CR123] Tucker CM, Eakin H, Castellanos EJ (2010). Perceptions of risk and adaptation: coffee producers, market shocks, and extreme weather in Central America and Mexico. Glob Environ Chang.

[CR124] USAID (2017) Country data sheets for coffee renovation and rehabilitation. Washington, DC. https://www.sustaincoffee.org/assets/resources/20171109_Country_data_sheets_vFinal.pdf Accessed 20 Oct 2020

[CR125] USDA FAS (United States Department of Agriculture Foreign Agricultural Service) (2019) Production, supply and distribution database. Washington, DC. https://www.fas.usda.gov/databases/production-supply-and-distribution-online-psd Accessed 20 Oct 2020

[CR126] USDA FAS (United States Department of Agriculture Foreign Agricultural Service) (2020a) Coffee annual Colombia. Bogota, Colombia. https://apps.fas.usda.gov/newgainapi/api/Report/DownloadReportByFileName?fileName=Coffee%20Annual_Bogota_Colombia_05-15-2020 Accessed 05 Oct 2020

[CR127] USDA FAS (United States Department of Agriculture Foreign Agricultural Service) (2020b) Coffee annual Costa Rica. San Jose, Costa Rica. https://apps.fas.usda.gov/newgainapi/api/Report/DownloadReportByFileName?fileName=Coffee%20Annual_San%20Jose_Costa%20Rica_05-15-2020 Accessed 02 Oct 2020

[CR128] Vaast P, Harmand JM, Rapidel B, Jagoret P, Deheuvels O (2016) Coffee and cocoa production in agroforestry—a climate-smart agriculture model. In: Climate change and agriculture Worldwide. Springer, Dordrecht, pp 209–224

[CR129] Valencia V, Naeem S, García-Barrios L, West P, Sterling EJ (2016). Conservation of tree species of late succession and conservation concern in coffee agroforestry systems. Agric Ecosyst Environ.

[CR130] Valencia V, García-Barrios L, Sterling EJ, West P, Meza-Jiménez A, Naeem S (2018). Smallholder response to environmental change: impacts of coffee leaf rust in a forest frontier in Mexico. Land Use Policy.

[CR131] van der Vossen HA (2005). A critical analysis of the agronomic and economic sustainability of organic coffee production. Exp Agric.

[CR132] van der Vossen H, Bertrand B, Charrier A (2015) Next generation variety development for sustainable production of arabica coffee (*Coffea arabica* L.): a review. Euphytica 204:243–256. 10.1007/s10681-015-1398-z

[CR133] VOA (2018) Latin America’s premium coffee growers branch out to cheaper beans. https://www.voanews.com/americas/latin-americas-premium-coffee-growers-branch-out-cheaper-beans Accessed 02 Oct 2020

[CR134] Volsi B, Telles TS, Caldarelli CE, Camara MRGD (2019). The dynamics of coffee production in Brazil. PLoS One.

[CR135] Ward R, Gonthier D, Nicholls C (2017). Ecological resilience to coffee rust: varietal adaptations of coffee farmers in Copán, Honduras. Agroecol Sust Food.

[CR136] Wiegel J, del Río M, Gutiérrez JF, Claros L, Sánchez D, Gómez L, González C, Reyes B (2020) Coffee and cacao market systems in the Americas: opportunities for supporting renovation and rehabilitation. International Center for Tropical Agriculture (CIAT). Cali, Colombia. https://www.researchgate.net/deref/https%3A%2F%2Fhdl.handle.net%2F10568%2F108108 Accessed 23 Oct 2020

