# Supplementary material for: Transformation of coffee-growing landscapes across Latin America. A review
Source: Agron Sustain Dev. 2021 Aug 31;41(5):62. doi: 10.1007/s13593-021-00712-0 (PMC8406019; doi:10.1007/s13593-021-00712-0)
Supplement: Supplementary file 1 — (DOCX 22 kb) [file 13593_2021_712_MOESM1_ESM.docx]

Supplementary Table 1. An illustrative list of biophysical changes in coffee farms and landscapes across northern and Andean Latin America, with examples at the national and local levels.

| Trend | | Examples | |
| --- | --- | --- | --- |
| 1.Replacement of traditional Arabica varieties by introgressed varieties that are resistant to coffee leaf rust | | National-level examples:   - Following the leaf rust outbreak in 2012, Guatemala replanted coffee farms with rust-resistant varieties at a large scale, renovating 15,860 ha by 2017 (Bunn et al. 2019). - In Peru, 40,000 ha of coffee had been renovated by 2016, primarily with Catimors which are resistant to coffee leaf rust. Another 40,000 ha are expected to be renovated by the end of 2020 (Romero 2020). - In Ecuador, an estimated 70% of the coffee area was renovated with new coffee varieties between 2013 and 2018 in response to the leaf rust outbreak (INEC 2019). - In Mexico, a combination of industry and government effort led to widespread renewal of coffee plantations with rust-resistant varieties (Amico et al. 2020). A total of 150,000 ha was rehabilitated in 2019, with plans for an additional 150,000 ha in 2020 (USDA FAS 2020h). - In Colombia, government-funded efforts to recover coffee plantations following the coffee leaf rust outbreak led to the replanting of 45% of the total coffee area (0.85 million ha) with resistant cultivars (van der Vossen 2015). - In Honduras, a massive government and industry-led renovation program greatly increased the area under resistant varieties, from 40% (in 2012, prior to the outbreak) to 62% in 2020 (Avelino and Anzueto 2020). - In Honduras, at least 201,000 ha had been renovated with rust-resistant varieties by 2015 (Wiegel et al. 2020).   Case studies:   - In Copan, Honduras, farmers have replaced traditional Arabica varieties with resistant, introgressed varieties. In 2012, prior to the leaf rust outbreak, 65% of coffee fields were composed of susceptible varieties; in 2015, following the outbreak, the percent of susceptible varieties had declined to 25% due to farmers renewing their plantations with introgressed resistant varieties (Ward et al. 2017). - In the La Sepultura region of Chiapas, Mexico, smallholder famers have replaced traditional Arabica varieties with introgressed resistant varieties in response to the coffee leaf rust outbreak (Valencia et al. 2018). | |
| 2. Conventional intensification of coffee production, involving a reduction in shade levels, increased use of agrochemicals and greater density of coffee bushes | | National-level examples:   - In Costa Rica, 10,800 ha were under traditional diverse shade in 1996; by 2012, all of this coffee had been converted to low diversity shade or open shade (Jha et al. 2014). - In Colombia, an estimated 37,502 ha of traditional, diverse shade was lost from 1996 to 2012 (Jha et al. 2014). - In El Salvador, an estimated 114,263 ha of traditional, diverse shade was lost from 1996 to 2012 (Jha et al. 2014). - In Guatemala, an estimated 20,450 ha of traditional, diverse shade was lost from 1996 to 2012 (Jha et al. 2014). - In Nicaragua, an estimated 18,049 ha of traditional, diverse shade was lost from 1996 to 2012 (Jha et al. 2014). - Tree cover within shaded coffee areas in El Salvador was lost at a rapid rate in the 1990’s and this trend continues to this day (Blackman et al. 2012). - In Guatemala, organic farmers shifted to conventional production methods after the coffee leaf rust outbreak and now use fungicides (Bielecki and Wingback 2019). - In Mexico, the renovation of coffee plantations with resistant varieties has led to a greater use of pesticides and inputs (Amico and Paz-Pellat 2018).   Case studies:   - In La Sepultura, Chiapas, Mexico, farmers have started applying fertilizers and chemicals to their previously-organic coffee plantations due to the adoption of new varieties that are resistant to coffee leaf rust (Valencia et al. 2018). Nineteen percent of farmers also removed shade trees in response to coffee leaf rust (Valencia et al. 2018). - In San Martin, Peru, 65% of smallholder coffee farmers reported having increased the use of organic and chemical inputs from 2014 to 2016, in order to control pests and disease (Jeezer et al. 2019). | |
| 3. Abandonment of coffee fields and conversion of coffee plots to other land uses | | National- level examples:   - In Guatemala, in areas with low altitude, flat terrain and close to existing croplands, coffee is being converted to croplands (Schmitt Harsh 2013). - Many of the larger coffee plantations in the lowlands of Guatemala have been replaced by rubber, oil palm and citrus plantations (Baumeister 2017). - In Peru, coffee farmers are abandoning their fields, resulting in a reduction in the total harvested area (USDA FAS 2020g). - In Colombia, the area under coffee has decreased in the traditional coffee triangle (particularly in the Valle del Cauca, Risaralda and Caldas), due to the expansion of avocado cultivation, other crops, tourism and urbanization (Rueda and Lambin 2013; Portafolio 2018).   Case studies:   - In the municipality of Zozocolco in Veracruz, Mexico, coffee agroforests were cleared and converted to pastures in response to the coffee crisis (Ellis et al. 2010). - In response to low coffee prices, coffee farmers in Emiliano Zapata in Central Veracruz, Mexico converted a portion of their coffee fields to sugarcane. Sugarcane has become an important source of household income for these farmers (Hausermann 2014). - From 1996 to 2003, 81.78% of the coffee agroforestry systems in Central Veracruz, Mexico, remained unchanged, 12.7% was converted to pasture or agriculture and 5.4% changes to urban areas (Hausermann 2014). - In Southern Guatemala, a survey of 50 farms showed a 35% loss in the area of coffee between 2000 and 2004 below 700 m, with coffee loss occurring on large and medium sized farms. Coffee was replaced by rubber and pastures in low-altitude farms and by bananas and timber plantations in mid-altitude farms (Hagar et al. 2013). - In the Chinantla subregion of Sierra Norte de Oaxaca, Mexico, 42% of the coffee farms were either abounded (35%) or converted to another land use (8%) during the decade from 1990 to 2010 (Hite et al. 2017). - Following the collapse of the coffee market, smallholder coffee farmers in Veracruz, Mexico, converted coffee to sugarcane or pasture, sold coffee plantations to urban developers, allowed coffee plantations to return to forest, and experimented with other cash crops (Eakin and Webbe 2009). - In the Costa Rican coffee district of Agua Buena, the proportion of farmland dedicated to coffee production diminished from 52% to 24% from 2000 to 2009, while the proportion of pasture land increased from 31% to 50% in response to low coffee prices. Only 24% of the land that was under coffee in 2000 was still in coffee in 2009 (Babin 2020). - In the coffee area of Turrialba, Costa Rica, there was a 50% reduction in the coffee area from 2000 to 2009, with coffee replaced by pastures, vegetable production, sugarcane or fruit trees and bananas (Bosselmann 2012). - In Guatemala, coffee is being converted to croplands in areas with low altitude, flat terrain and close to existing croplands, (Schmitt Harsh 2013). | |
| 4. Expansion of coffee into forested areas, leading to deforestation | | National-level data:   - In Honduras, coffee production has expanded at the expense of natural forests (Bunn et al. 2017). - Coffee cultivation is expanding into the Amazonian region of Ecuador and Peru, contributing to deforestation (Somarriba and Sampson 2018). - Expansion of coffee production has been an important factor driving forest loss in Guatemala, where the area under forest was reduced from 44% to 33% of total land cover between 1990 and 2015 (Bunn et al. 2018).   Case studies:   - In the San Martin region of Peru, the area under coffee production tripled to an estimated area of 102,101 ha between 1995 and 2010, at the expense of primary forest (Marquadt et al. 2019). - Coffee expansion was positively correlated with deforestation in 8 Amazonian departments in Peru, with Cajamarca, Ucayali, and Junin having the strongest correlations (Ganzenmuller and Castro-Nunez 2019). - In the transboundary Trifinio region (which spans Honduras, Guatemala and El Salvador), an analysis of forest and land-use change found that the loss of forest cover was positively correlated with coffee expansion. From 1983 to 2010, forest cover declined from 3,300 km^2^ to 2,302 km^2^, while the area under coffee went from being virtually non-existent (<0.1% of the total area) to covering over 75 km^2^ (Schlesinger et al. 2017). - In the municipalities of El Provenir, Ángel Albino Corzo, Siltepec and the upper areas of La Concordia in Chiapas, Mexico, coffee production is leading to deforestation and degradation (Covaleda et al. 2014). - In La Sepultura, Chiapas, 17% of coffee farmers indicated they planned to establish new coffee fields (with high yielding, resistant varieties) in existing forest areas and 73% planned to expand coffee into existing fallow areas (Valencia et al. 2018). | |
| 5. Introduction of Robusta into new areas where coffee was previously not grown | | National patterns:   - In Guatemala, Anacafé (the country’s coffee institute) is actively encouraging the establishment of Robusta coffee in altitudes below 800m, where coffee leaf rust has made Arabica farming unprofitable (Nicolson and Menchu 2018), and in elevations that are no longer ideal for Arabica coffee production due to warming temperatures (VOA 2018). Anacafe aims to increase the country's Robusta harvest fourfold to 300,000 60-kg bags (VOA 2018). - In Nicaragua, Nestle and Mercon (a long-term exporter of coffee from Nicaragua) expect the country’s robusta crop to increase by more than 6 times (to 238,000 bags) by 2025 (VOA 2018). - National bans on the production of Robusta coffee were lifted in Nicaragua (2013) and Costa Rica (2018), opening these countries to potential Robusta production (Pretel 2018). - In Mexico, the government is supporting the establishment of 20,000 ha of Robusta coffee cultivation in the southern and southeastern states of Chiapas, Veracruz and Tabasco (Aceves Navarro et al. 2018).   Case studies:   - Robusta coffee is being introduced to the Autonomous Region of the South Atlantic of Nicaragua in areas where coffee was not previously grown (Nicolson and Menchu 2018). - In Nueva Guinea, Nicaragua, subsistence farmers are now actively converting degraded pasture areas into Robusta production, with more than 900 ha of Robusta coffee planted in 2016 (Bjørge 2017). - In Mexico, the government and private institutions are promoting the expansion of Robusta coffee plantations primarily in areas under 800 masl, but also among communities of the Pacific slope of the Sierra Madre mountain range (Amico et al. 2020). - In southern Chiapas, Mexico, the replacement of shaded Arabica coffee by Robusta with little or no shade is occurring in steep, mountainous areas that are still suitable for shade-grown Arabica coffee (Barrera 2016). - Robusta coffee is being tested in parts of Colombia by the Colombian Cooperation for Agricultural Research, AGROSAVIA (Portafolio 2017). | |
| 6. Urbanization of coffee areas | | National-level examples:   - The area under coffee in Costa Rica declined 20% from 2001 to 2008 due to a combination of urbanization, pasture production and pineapple cultivation (Virginio Filho and Abarca 2008). - Increased land prices have led to the urbanization of coffee lands in Costa Rica (Jha et al. 2014). - Most of the loss of coffee agroforestry systems in El Salvador between 1990 and 2000 was due to urbanization. Urbanization accounted for 90% of the loss of coffee in the western region of the country and 68% of the clearing in the central region (Blackman et al. 2012).   Case studies:   - Coffee agroforests in Veracruz, Mexico are being replaced by urban development (Hausermann 2014). From 1996 to 2003, in a case study in Central Veracruz, Mexico, 81.8% of the coffee AFS remained unchanged, 12.7% was converted to pasture or agriculture and 5.4% changed to urban areas (Hausermann 2014). - The area under coffee production in the traditional coffee triangle (‘eje cafetero’) of Colombia has declined between 2000 and 2018, in part due to urbanization. The area under coffee in Pereira has fallen from a high of 15,000 ha to 4,000 ha, due to urbanization (Portafolio 2018). | |
| 7. Increase in the coffee area grown under voluntary sustainability standards, with related changes in shade levels and/or on-farm forest cover | | Case studies:   - In Santander, Colombia, Rainforest Alliance-certified coffee farms had greater tree cover and greater landscape connectivity than non-certified farms (Rueda et al. 2015) - In Sander, Colombia, Rainforest Alliance-certified coffee farmers were more likely to plant trees and protect water sources through reforestation than non-certified farmers (Reuda and Lambin 2013b) - Organic farms in Costa Rica, Guatemala and Honduras had greater farm-level tree species richness and diversity than conventional farms. They also had higher shade levels and more tree strata (Haggar et al. 2015). | |
